# Decoding the Role of Epigenetics in Breast Cancer Using Formal Modeling and Machine-Learning Methods

**DOI:** 10.3389/fmolb.2022.882738

**Published:** 2022-07-11

**Authors:** Ayesha Asim, Yusra Sajid Kiani, Muhammad Tariq Saeed, Ishrat Jabeen

**Affiliations:** School of Interdisciplinary Engineering and Sciences (SINES), National University of Sciences and Technology, (NUST), Islamabad, Pakistan

**Keywords:** RUNX3 signaling pathway, Dnmt1, machine learning, qualitative modeling, c-myc, SMBioNet

## Abstract

Breast carcinogenesis is known to be instigated by genetic and epigenetic modifications impacting multiple cellular signaling cascades, thus making its prevention and treatments a challenging endeavor. However, epigenetic modification, particularly DNA methylation-mediated silencing of key TSGs, is a hallmark of cancer progression. One such tumor suppressor gene (TSG) *RUNX3* (Runt-related transcription factor 3) has been a new insight in breast cancer known to be suppressed due to local promoter hypermethylation mediated by DNA methyltransferase 1 (DNMT1). However, the precise mechanism of epigenetic-influenced silencing of the *RUNX3* signaling resulting in cancer invasion and metastasis remains inadequately characterized. In this study, a biological regulatory network (BRN) has been designed to model the dynamics of the DNMT1–*RUNX3* network augmented by other regulators such as p21, c-myc, and p53. For this purpose, the René Thomas qualitative modeling was applied to compute the unknown parameters and the subsequent trajectories signified important behaviors of the DNMT1–*RUNX3* network (i.e., recovery cycle, homeostasis, and bifurcation state). As a result, the biological system was observed to invade cancer metastasis due to persistent activation of oncogene c-myc accompanied by consistent downregulation of TSG *RUNX3.* Conversely, homeostasis was achieved in the absence of c-myc and activated TSG *RUNX3*. Furthermore, DNMT1 was endorsed as a potential epigenetic drug target to be subjected to the implementation of machine-learning techniques for the classification of the active and inactive DNMT1 modulators. The best-performing ML model successfully classified the active and least-active DNMT1 inhibitors exhibiting 97% classification accuracy. Collectively, this study reveals the underlined epigenetic events responsible for *RUNX3*-implicated breast cancer metastasis along with the classification of DNMT1 modulators that can potentially drive the perception of epigenetic-based tumor therapy.

## 1 Introduction

Breast cancer is one of the frequently diagnosed lethal malignancies affecting millions of women worldwide. The risk factors include both genetic and epigenetic abnormalities whereby the latter provides early prognostic biomarkers for breast cancer therapeutics (Chen, 2012; Sun et al., 2017). Furthermore, DNA methylation is the most prominent epigenetic marker that frequently influences the expression or silencing of genes involved in vital cellular activities such as cell proliferation, programmed cell death, and cell differentiation (Chen, 2012). DNA methylation is mediated by DNA methyltransferases (DNMTs), in which the DNMT1 isomer is explicitly responsible for the maintenance of methylation status during replication (S-phase) (Pouliot et al., 2015). Briefly, it transfers the methyl group (−CH3) on cytosine in CpG dinucleotide regions by keeping the CpG islands non-methylated that are preferably located in the proximal promoter region of a gene (Tang et al., 2009). On the contrary, the hypermethylation of promoter CpG islands of TSGs due to the overexpression of DNMT1 is a popular mechanism of gene silencing and a hallmark of cancer (Subramaniam et al., 2014; Pouliot et al., 2015).

DNMT1-regulated hypermethylation of the TSG *RUNX3* promoter region is a new insight and an early event in breast cancer predominantly in TNBC (triple-negative breast cancer) (Widschwendter and Jones, 2002; Jung et al., 2007; Jiang et al., 2008; Subramaniam et al., 2009; Shin et al., 2016). Generally, *RUNX3* is known to elicit its tumor-suppressive ability through major cancer signaling pathways including TGF-β, Wnt/β-catenin, and KRAS (Chen, 2012). *RUNX3* either influences the downstream target of tumor suppressor signaling pathways or acts as an antagonist for oncogenic pathways to exert its antitumor activity. However, despite the evident role of *RUNX3* in breast cancer and the relation of DNMT1 with *RUNX3* gene, no study has explored the underlying epigenetic molecular events by which DNMT1 silences *RUNX3* to promote breast cancer metastasis.

Furthermore, due to the pivotal implication of DNMT1 in various tumors (Li et al., 2019) and the reversible nature of its methylation activity, targeting DNMT1 through small modulators is a promising pharmacological intervention to revive the suppressed TSGs (such as *RUNX3*). To date, two DNMT inhibitors (i.e., azacytidine and decitabine) have been approved by FDA for the treatment of myeloid malignancies (Gnyszka, Jastrzębski, and Flis 2013). However, the potential side effects associated with these drugs limit their use in high-grade malignancies. Therefore, the development of more potent DNMT1 inhibitors acquiring potential binding pocket features is highly desirable to restore the suppressed TSGs (*RUNX3*) in cancer therapeutics. Previously, several *in vivo* and computational studies have targeted DNMT1 for therapeutic purposes (Yoo 2011; Yoo, Kim, and Robertson 2012; Mirza et al., 2013; Krishna et al., 2017); however, its impact on downstream target genes, particularly *RUNX3,* requires further elucidation.

**FIGURE 1:**
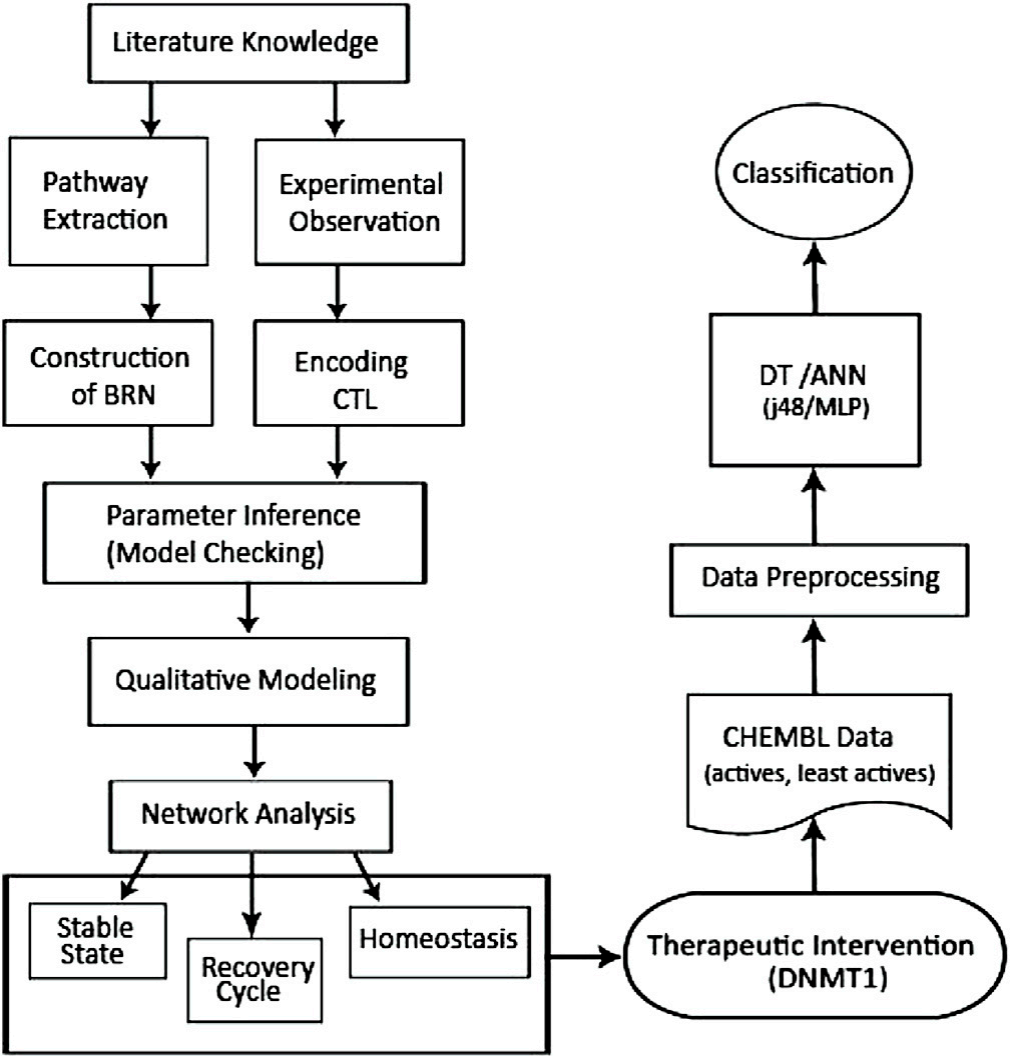
Flowchart showing the overall methodology utilized in the current study. Qualitative modeling of the BRN to explore and model the DNMT1–*RUNX3* network was followed by the application of ML techniques to construct the predictive ML (DT and ANN) models using the DNMT1 data set extracted from the ChEMBL database.

Therefore, in the present study, a BRN of the DNMT1–*RUNX3* signaling was developed to provide an insight into the epigenetic-mediated silencing of *RUNX3* leading to cancer development and metastasis. For this purpose, qualitative modeling by the René Thomas formalism was applied in a SMBioNet tool utilizing the existing wet-laboratory data in the form of computation tree logic (CTL) for parameter estimation and verification through the model checking technique (Figure 1). The model trajectories were explored to identify the paths involved in the overexpression of DNMT1, activation of oncogene (c-myc), and suppression of tumor suppressor genes (*RUNX3*, p21, and p53) leading to cancer invasion or recovery (homeostasis). In addition, DNMT1 was advocated as a potential drug target and favored to be subjected to ML approaches to classify diverse DNMT1 modulators. Decision tree (DT) and neural network classifiers were built utilizing the data set of DNMT1 from the ChEMBL database to identify potential two-dimensional binding features essential for the modulation of DNMT1 activity (Figure 1).

## 2 Materials and Methods

The overall methodology used in the current study is provided in Figure 1.

### 2.1 Qualitative Modeling Framework

Definition 1 Biological Regulatory Network (BRN)

A BRN is a labeled, directed graph G = (V, E) in which biological regulators are denoted as a set of nodes or vertices V and their interactions as a set of edges E⊆ V × V. Each edge is labeled by a pair of elements (*τ, σ*), where *τ* is the threshold at which a gene U starts regulating a gene V, and *σ* represents the type of interactions *σ* = + (activation) and − (inhibition) between the nodes (Bernot et al., 2004).

Definition 2 Qualitative State

The state of the BRN is a configuration of the expression level of all the nodes at a particular time instant. A state is n-tuple S = {s_v1_ , . . . , s_vn_}, ∀s_vi_ ∈ δ_vi_, where s_vi_ is the expression level of _vi_ (Bernot et al., 2004).

Definition 3 Resources

Each *vi* in a state of the BRN is controlled by its precursors G^**-**^_vi_, called the set of resources ω_vi_.

Let G = (V, E), at level s_vj_, ω_vi_, defined as:
ω_vj_ = vi ∈ G^−^_vj_ ∣ (s_vi_ ≥ τ_vi_,_vj_ and σ_vi,vj_ = +) or (s_vi_ < τ_vi,vj_ and σ_vi,vj_ = −) (Bernot et al., 2004).

Definition 4 Parameters

Parameters of a biological regulatory network are indexed by its set of resources. It is a cartesian product of each variable’s element and resource (Saeed et al., 2016).

Definition 5 Betweenness Centrality

Network analysis techniques such as graph theory–based approaches can be applied to further analyze the state graph by sorting it based on betweenness centrality (Freeman 2019). The betweenness centrality measures the extent to which a single vertex or node is more central/connected than all other nodes. For a particular node, the centrality metric is measured with the number of shortest paths that pass through it, whereas the betweenness would be high for a particular node if it appears in many shortest paths (Golbeck 2015). Likewise, the qualitative states with higher betweenness centrality value are more likely to occur in the system, implying that these entities are frequently expressed in biological phenomena. Moreover, the central or more connected nodes in the system might represent a potential therapeutic target (Golbeck 2015; Saeed et al., 2016).

### 2.2 Qualitative Modeling and Parameter Estimation

René Thomas introduced a graph theory–based approach for qualitative modeling of dynamic biological regulatory networks (Thomas 1978). In this method, each BRN (Definition 1) is modeled as a weighted, directed graph, which consists of a set of nodes and edges. Nodes represent a biological entity (i.e., genes or proteins), whereas edge represents the relationship of activation or inhibition between the nodes.

Herein, the role of the epigenetic (DNMT1)-mediated *RUNX3* silencing in cancer development and progression was examined by regulating key oncogenes and TSGs. The literature-driven information was used to generate the BRN, which signifies important entities involved in the DNMT1–*RUNX3* signaling and the relationship (activation and inhibition) among the chosen entities. The unknown parameters were inferred by encoding wet-laboratory biological observations as propositional calculus or more precisely computation tree logic (CTL) and verified through the model checking technique as previously reported by Saeed et al. (2018). Briefly, model checking is an automatic technique based on the exhaustive exploration of the entire state space of a biological system, which therefore allows the analysis and cross-verification of a large number of possible outcomes of a network (Bernot et al., 2004). All compatible combinations of parameters (Definition 4) were generated and evaluated by a model checker using a SMBioNet tool, and the model parameters that violate laboratory data were eliminated.

Briefly, SMBioNet (Khalis et al., 2009) is a qualitative framework–based tool that calls New Symbolic Model Verifier (NuSMV) (Cimatti et al., 2002) as a model checker. NuSMV works by considering a Model M of the BRN and its property *φ*, which exhaustively explores M to verify *φ.* The SMBioNet engages in this principle to identify logical parameters of the models that comply more with the known observations. Once the computational verification with laboratory data was completed, the resultant model and all its trajectories were further analyzed to understand how the systematic evolution of the DNMT1–*RUNX3* system takes place with time. The network was explored using the concept of betweenness centrality to underline important trajectories of the dynamic biological system (i.e., homeostasis, bifurcation state, and recovery cycle). Furthermore, the paths involved in the activation of oncogenes, suppression of tumor suppressor genes, cancer invasion, and recovery were also identified. This study also highlighted a qualitative bifurcating at which the system can invade tumorigenesis or normal homeostasis depending upon the successive changes in the expression level of TSG and oncogenes.

### 2.3 Data Set Compilation or Collection

A total of 738 DNMT1 inhibitors were collected from the ChEMBL database (target ID ChEMBL1993). Only those compounds were extracted for which the biological activity was estimated experimentally as the inhibitory potency (IC_50_ value). Initially, the removal of duplicates, small fragments (MW < 200), and inconsistent activity values (IC_50_) was performed, which was followed by the exploration and manual correction of stereoisomers. The data preprocessing resulted in a final data set of 242 DNMT1 inhibitors with IC_50_ values in the range of 0.01–1,600 μM (Section 2, Supplementary Tables S1, S2).

Briefly**,** our data set contains compounds of diverse origin that include natural, synthetic (nucleoside/non-nucleoside), and FDA-approved drugs against DNMT1, thus incorporating all major scaffolds of DNMT1 inhibitors available to date. The shortlisted DNMT1 inhibitors were used to build machine-learning (DT and ANN) models. Therefore, a diverse subset selection approach was utilized to divide the data set into a training set (80%) and a test set (20%) for model building and validation, respectively. Briefly, a diverse subset splits the data into two sections based on chemical diversity calculated as a function of distance between molecular descriptors (Koutsoukas et al., 2013). The absolute biological activity values were converted into binary numbers on the basis of an activity threshold value (IC_50_ ≤ 10 µM) such that 1 represents active and 0 indicates least-active class of DNMT1 inhibitors. This binarization of DNMT1 data was supported by a histogram plot provided in Supplementary Figure S1.

### 2.4 Calculation and Selection of 2D Chemical Descriptors

All 2D MOE descriptors (2019.01) (ULC 2018) by excluding energy-related descriptors were calculated for the training set. A total of 158 2D descriptors were computed through MOE after which the redundant, null, and constant value descriptors were excluded from the final set. The descriptors with negligible relevancy and weightage were also removed from the final set of descriptors to improve the overall predictive ability of ML models. Consequently, the selected descriptors were provided as an input to WEKA (3.9.3) for the construction of a DT classifier that was further used for shortlisting of the most relevant and decision-making attributes.

### 2.5 Machine-Learning Approaches

Herein, the DT and ANN classification models were built using the training set and the models were validated using the 20% test set compounds.

#### 2.5.1 Decision Tree

The C4.5 variant of the J48 algorithm implemented in WEKA was used to build the univariate tree of training set attributes. The J48 algorithm works by splitting the data into smaller subsets based on features that will produce the most uniform child node at each step (Yosipof, Guedes, and García-Sosa 2018). The process is repeated iteratively until no more splits can be made or data are uniformly classified into terminal nodes. Tree parameters were tuned to improve the overall performance of the model and limit the overfitting of data. Therefore, the lowest number of confidence factor was used to incur more pruning and a minimum of one instance per leaf was set for the splitting rule, with a 10-fold cross-validation approach.

#### 2.5.2 Artificial Neural Network

Artificial neural networks (ANNs) are nonparametric human nervous system–inspired computational models, which process complex input information to produce the output. A multilayer perceptron (MLP) function of WEKA (3.9.3) was utilized to build the ANN model of DNMT1 inhibitors. The MLP network typically consists of at least three layers: one input, one output, and one hidden layer (can be more than one). The perceptron calculates a linear combination of inputs and their weights to compute a sum, and the output is calculated through an activation function (most often the sigmoid) (Jiao et al., 2020). Moreover, MLP uses back-propagation to find the optimized input weights and builds hidden layer(s) for the classification of nonlinear data (Frank, Hall, and Witten 2017). Generally, MLP normalizes each attribute by default, to improve the performance of the network. Overall, model training was performed using a 10-fold cross-validation and the parameters were optimized on the basis of number and nodes of hidden layer by constructing several perceptron models. The default values of momentum, learning rate and training time were used in order to build the best-fit neural network.

#### 2.5.3 Model Performance Validation

The DT and ANN models were trained using the training set (80%), and the classification was validated using the test set (20%). The performance of the machine-learning model was further evaluated using statistical parameters including accuracy (Eq. 1), sensitivity (Eq. 2), specificity (Eq. 3), F-measure (Eq. 6), and MCC measure (Eq. 7). Accuracy is the percentage of correctly classified active and least-active compounds, whereas specificity is the percentage of true least-active predicted compounds, and sensitivity is the proportion of true active compounds predicted from our model. In addition, F-score, also known as balanced accuracy, measures the precision (how many compounds are correctly classified) and robustness (resistance to errors) of ML models. Likewise, Matthew’s correlation coefficient (MCC) measures the difference between actual and predicted values, and it is only high (near to 1) if all the classes are predicted in good proportion.

Overall accuracy:

$$\frac{\mathrm{TP}+\mathrm{TN}}{\mathrm{TP}+\mathrm{FP}+\mathrm{TN}+\mathrm{FN}}.$$ (1)

Sensitivity (true-positive rate):

$$\mathrm{Tp}=\frac{\mathrm{TP}}{\mathrm{TP}+\mathrm{FN}}.$$ (2)

Specificity (true-negative rate):

$$\mathrm{Tn}=\frac{\mathrm{TN}}{\mathrm{TN}+\mathrm{FP}}.$$ (3)

Precision:

$$\frac{\mathrm{TP}}{\mathrm{TP}+\mathrm{FP}}.$$ (4)

Recall:

$$\frac{\mathrm{TP}}{\mathrm{TP}+\mathrm{FN}}.$$ (5)

F-measure:

$$2\times \frac{\mathrm{precision}\times \mathrm{recall}}{\mathrm{precision}+\mathrm{recall}}.$$ (6)

Mathew’s correlation coefficient:

$$C=\frac{\mathrm{TP}\times \mathrm{TN}-\mathrm{FP}\times \mathrm{FN}}{\sqrt{\left(\mathrm{TP}+\mathrm{FP}\right)\left(\mathrm{TP}+\mathrm{FN}\right)\left(\mathrm{TN}+\mathrm{FP}\right)\left(\mathrm{TN}+\mathrm{FN}\right)}}.$$ (7)

## 3 Results

**FIGURE 2:**
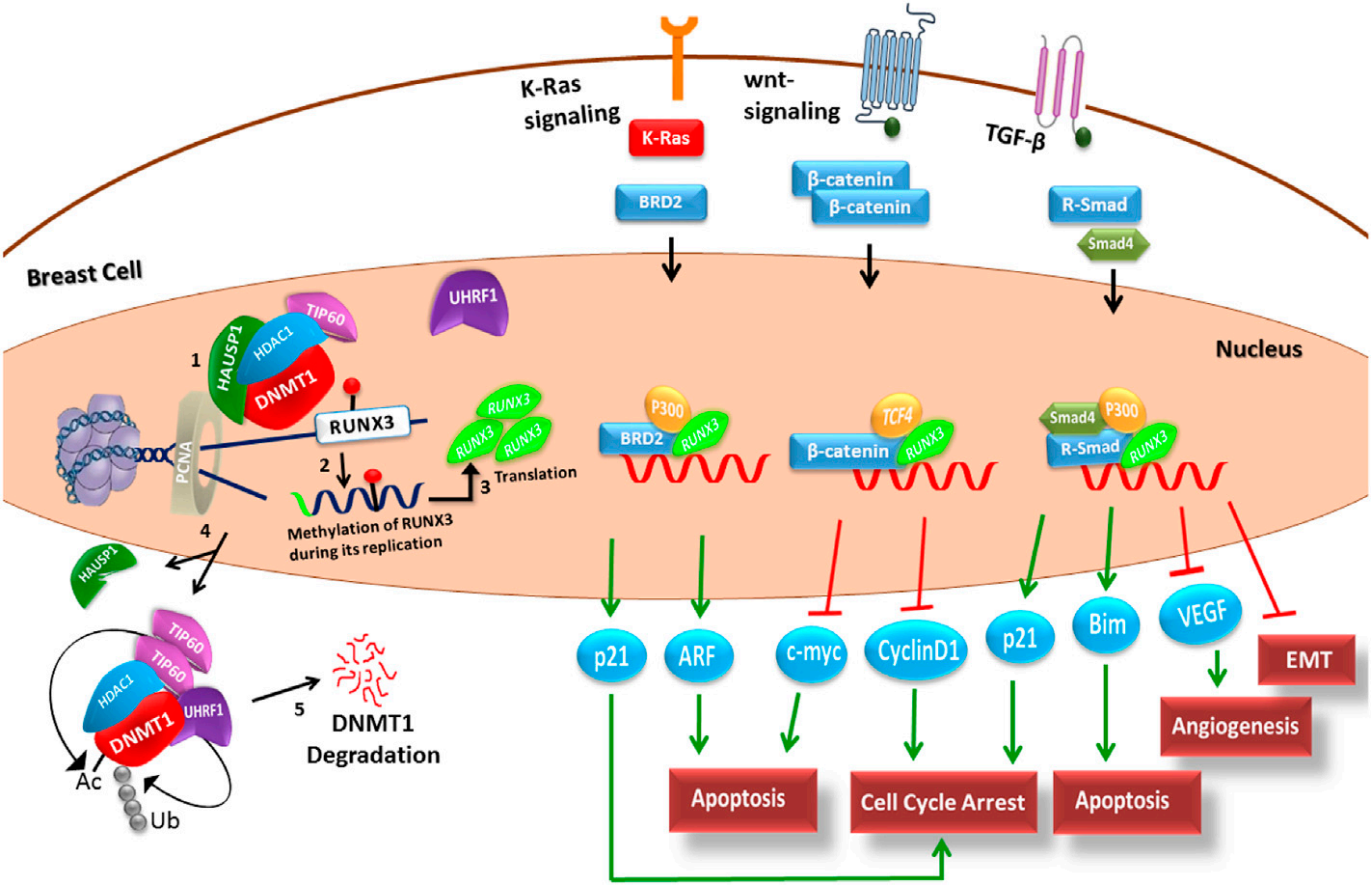
Schematic knowledge-based network is presented to illustrate the stepwise process of replication and methylation (by DNMT1) of TSG *RUNX3* and its implication in different cancer pathways. Step 1: At the replication fork, UHRF1 recognizes hemimethylated DNA and recruits other proteins including DNMT1, Tip60, HAUSP1, HDAC1, and PCNA to make a macroprotein complex. Step 2: DNMT1 in complex with these regulators transfers a methyl group through the base flip mechanism onto the nascent daughter strand. The green color highlighted in the daughter strand depicts the hypomethylated promoter region of the nascent *RUNX3* gene. The red-headed lollipop structure here mimics the normal methylation status of *RUNX3* gene. Step 3: Transcription machinery then successfully identifies the promoter region to translate the functional *RUNX3* protein, which acts as a tumor suppressor and combats the cancerous environment through the regulation of major signaling pathways including TGF-beta, Wnt/β, and KRAS pathways. TSG *RUNX3* exerts its antitumor activity by regulating the transcription of significant target genes including p21, c-myc, and p53 (oval blue structures at the bottom). Steps 4 and 5: The entire macroprotein complex after performing its function undergoes stepwise proteasomal degradation in the late S-phase of cell cycle. PCNA = proliferating cell nuclear antigen; DNMT1 = DNA methyltransferase 1; HAUSP1 = herpesvirus-associated ubiquitin-specific protease; HDAC1 = histone deacetylase1; Tip60 = histone acetyltransferase; UHRF1 = ubiquitin-like, containing PHD and RING finger domains 1; Ac= acetylation; *RUNX3* = Runt-related transcription factor 3; TGF-β = transforming growth factor-beta; VEGF = vascular endothelial growth factor; EMT = epithelial–mesenchymal transition; and TSG = tumor suppressor gene.

A schematic model depicting the stepwise process of replication and methylation of TSG *RUNX3* by DNMT1 and its subsequent influence on various cancer signaling pathways was established using the literature-driven information. Qualitative modeling was then performed to unfold the cellular events involved in the epigenetic, that is, DNMT1-mediated silencing of *RUNX3* that leads to cancer invasion. The comprehensive literature-driven pathway of DNMT1–*RUNX3* is elaborated in the Supplementary Material Section S3, Figure 2. Briefly, a series of stepwise events at the replication fork led by macroprotein complexes (DNMT1, UHRF1, HAUSP, Tip60, HDAC1, and PCNA) ensures the methylation of the newly synthesized daughter strand during the S-phase (Figure 2). As a result, the nascent *RUNX3* strand acquires the normal status of methylation, whereas the promoter region remains hypomethylated to facilitate active transcription of *RUNX3* gene. Subsequently, *RUNX3* (transcription factor) functions as a tumor suppressor protein to combat cancer initiation and metastasis through various signaling pathways. The TSG *RUNX3* elicits an antitumor activity by regulating the transcription of target genes (p21, c-myc, etc.) of the major cancer signaling cascades, such as transforming growth factor-beta (TGF-β), Wnt/β-catenin, and mitogenic KRAS pathway as explained in Figure 2.

**FIGURE 3:**
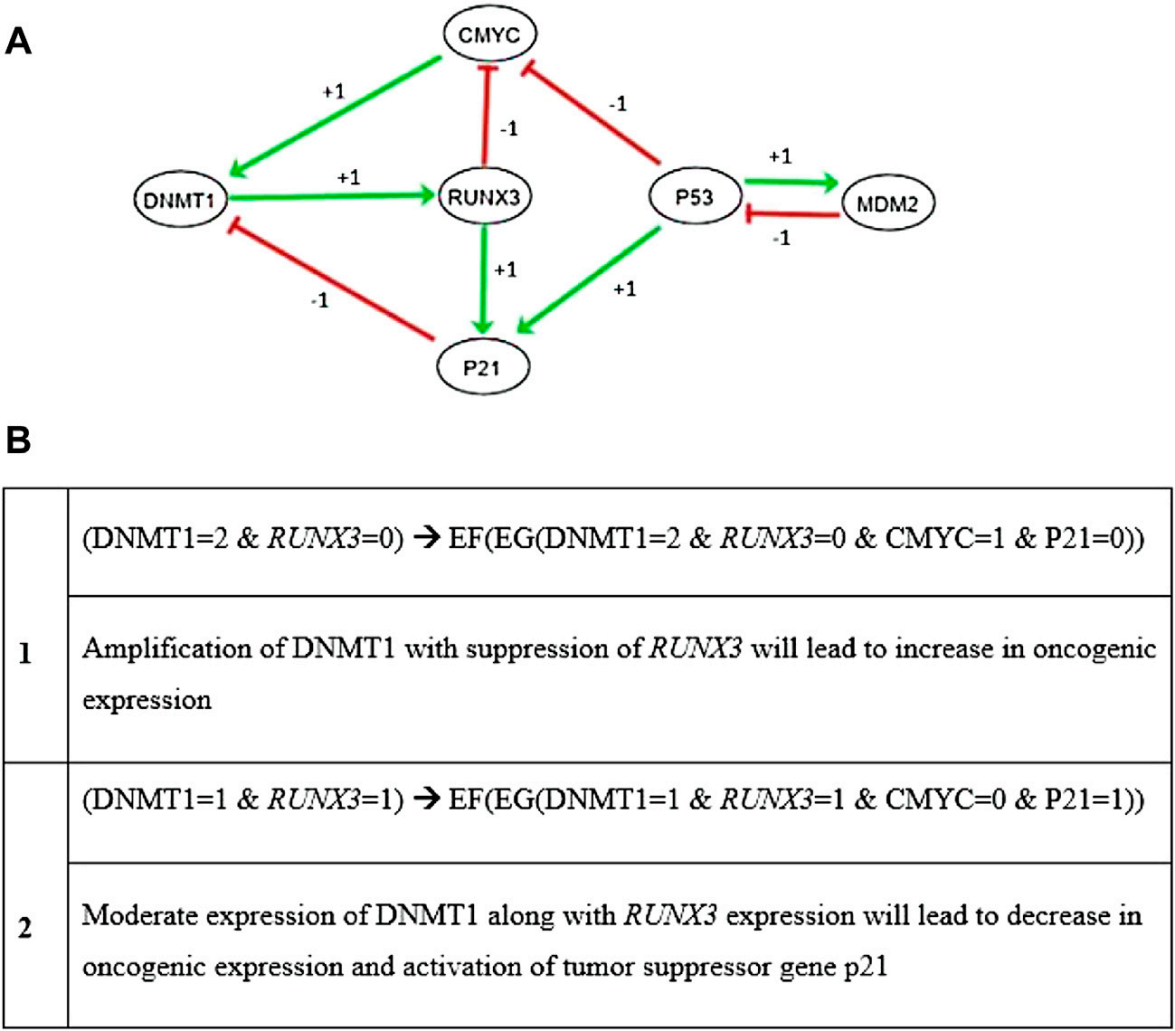
**(A)** DNMT1–*RUNX3* interaction graph (BRN) was generated by utilizing the preferred entities to discover all the important activation (+) and inhibition (−) relationships among them. The network demonstrates predominantly two oscillatory behaviors in DNMT1–*RUNX3* graph. The first one illustrates the *RUNX3*-stimulated onset of p21, which in turn positively regulates DNMT1 (shown with red arrows). The other loop exhibits the inhibition of c-myc by *RUNX3*, which also instigates the onset of DNMT1 (shown with green pointed arrows). In addition, p53 can also be seen regulating DNMT1 through the activation of p21 and inhibition of c-myc signals. **(B)** The second part of the figure demonstrates two CTL observations utilized by SMBioNet for the estimation of parameters that were later used to generate the state graph of the dynamic model. According to CTL formulas, the overexpression of DNMT1 is responsible for hypermethylation at the promoter region of *RUNX3* and ultimately its suppression, which is associated with many cancer types including breast cancer. Each circle/node represents a gene, and the arrows among them show the type of interaction they hold. Activation is denoted with green pointed arrows, and blunt red arrows represent inhibition whereby the weight of the arrows depicts threshold values of interactions.

From literature-driven pathways described in Figure 2, a qualitative biological regulatory network was generated based on preferred entities. DNMT1, *RUNX3*, p21, c-myc, p53, and MDM2 nodes were selected due to their well-established functionality in the biological system (Supplementary Material Section S3). As a result, the BRN (Figure 3A) composed of total six nodes and nine interactions exhibiting all the significant activation and inhibition relationships was obtained. Initially, negative feedback loops were observed from an inhibitory set of genes required by the system to generate the stable states. According to the interaction graph (Figure 3A), *RUNX3* transactivates p21 to maintain the concentration of DNMT1 through a negative feedback loop. In addition, *RUNX3* inhibits the onset of oncogene c-myc that might upregulate the expression level of DNMT1 in a positive manner. Interestingly, p53 also prevents upregulation of DNMT1 through the activation of p21 and inhibition of oncogene c-myc. Furthermore, the proposed network characterized oscillatory behavior of two regulatory loops involving DNMT1 and *RUNX3*: 1) a negative feedback loop between p21 and DNMT1 through *RUNX3* and 2) a positive feedback loop between c-myc and DNMT1 through *RUNX3* (Figure 3A)*.* It is notable that the representation of the BRN as a weighted, directed graph (Figure 3A) was obtained using GENOTECH through the implementation of discrete formalism. Therefore, the interaction graph utilized the qualitative data only, that is, type of interaction (activation +, or inhibition −) among the nodes (genes), and the threshold value for each interaction. However, modeling the dynamic behavior of such complex systems that include both positive and negative loops requires the computation of accurate logical parameters. These parameters were generated in the SMBioNet software using the known experimental observations encoded as CTL formulas reported in Figure 3B. The first CTL observation searches for a state with high expression of DNMT1 and oncogenes leading to tumor invasion. The later CTL formula seeks for the stable state or homeostasis that has normal expression level of tumor suppressor genes (Figure 3B). These observations were coded as input in SMBioNet to generate the preferred set of logical parameters.

### 3.1 Parameters

**FIGURE 4:**
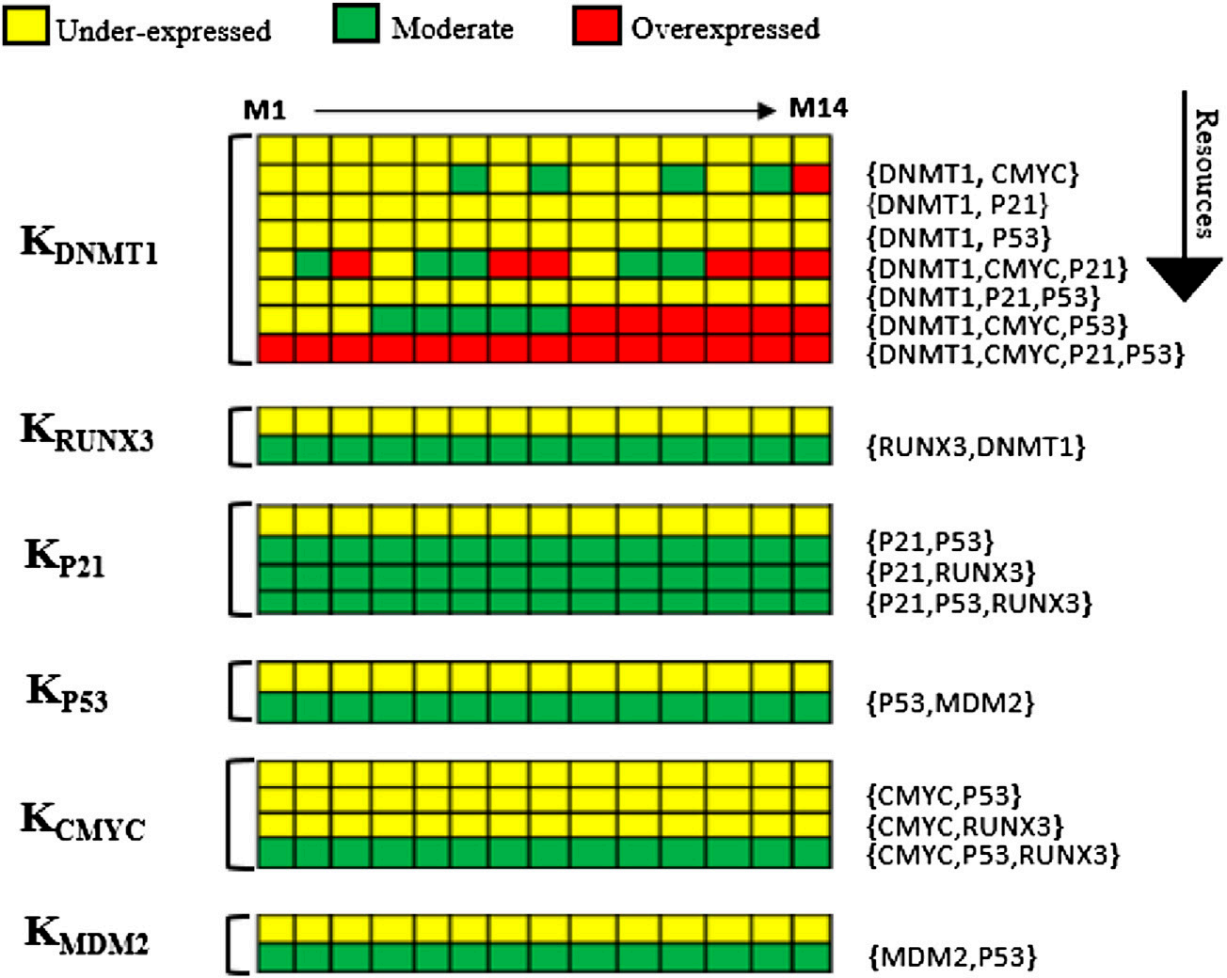
Heat map of logical parameters computed on SMBioNet shows 14 distinct sets of parameters. A preferred set of parameters were estimated through model checking rendered as heat map along with their resources (M1–M14). Each column represents a distinct set of logical parameters where a moderate expression of an entity is expressed using green color, an overexpression is expressed using red color, and an underexpression is illustrated using yellow color in the heat map.

SMBioNet computed a total of 14 sets of logical parameters that are presented as a heat map in Figure 4. The preferred set of logical parameters verified through model checking might reflect the probable biological trajectories in cancer invasion and recovery. According to the known biological observations, the most significant change occurs in the expression of DNMT1 and *RUNX3* in different types of malignancies including breast tumor. The heat map suggests eight critical resources of DNMT1 based on the presence and absence of its activators and inhibitors. For instance, (CMYC) indicates the presence of c-myc, and (CMYC, P21) represents the presence of activator c-myc and the absence of inhibitor p21. Likewise, (CMYC, P21, and P53) shows the presence of activator c-myc whereby no inhibitor is present in the system. The ability of each entity to evolve is described as a function of the presence or absence of its resource as shown in Figure 4.

The trend of DNMT1 being induced in the presence of c-myc was observed in all 14 parameter sets. Therefore, a parameter set that allows DNMT1 to achieve its maximum threshold value of “2” was selected to generate the state graph, assuming that this concentration is lethal for breast cells. The selected parameters (M6) allow all nodes to interact with others corresponding to the natural dynamic phenomenon, while maintaining their interdependencies for the activation or suppression. The source code of input models is provided in the Supplementary Material Section S1. The calculated parameters indicate that DNMT1 maintains a higher expression level in the presence of oncogene c-myc signaling. Conversely, an activation of an inhibitor p21 or p53 prevents the expression level of DNMT1 to exceed the normal threshold value; it is also observed that TSG *RUNX3* is activated in the presence of DNMT1 signal. The expression of p53 is increased when the MDM2 inhibition signals are absent in the system. However, the collective behavior of the genes in a dynamic biological system can only be concluded by interpreting the trajectories in the state transition graph.

### 3.2 State Graph

**FIGURE 5:**
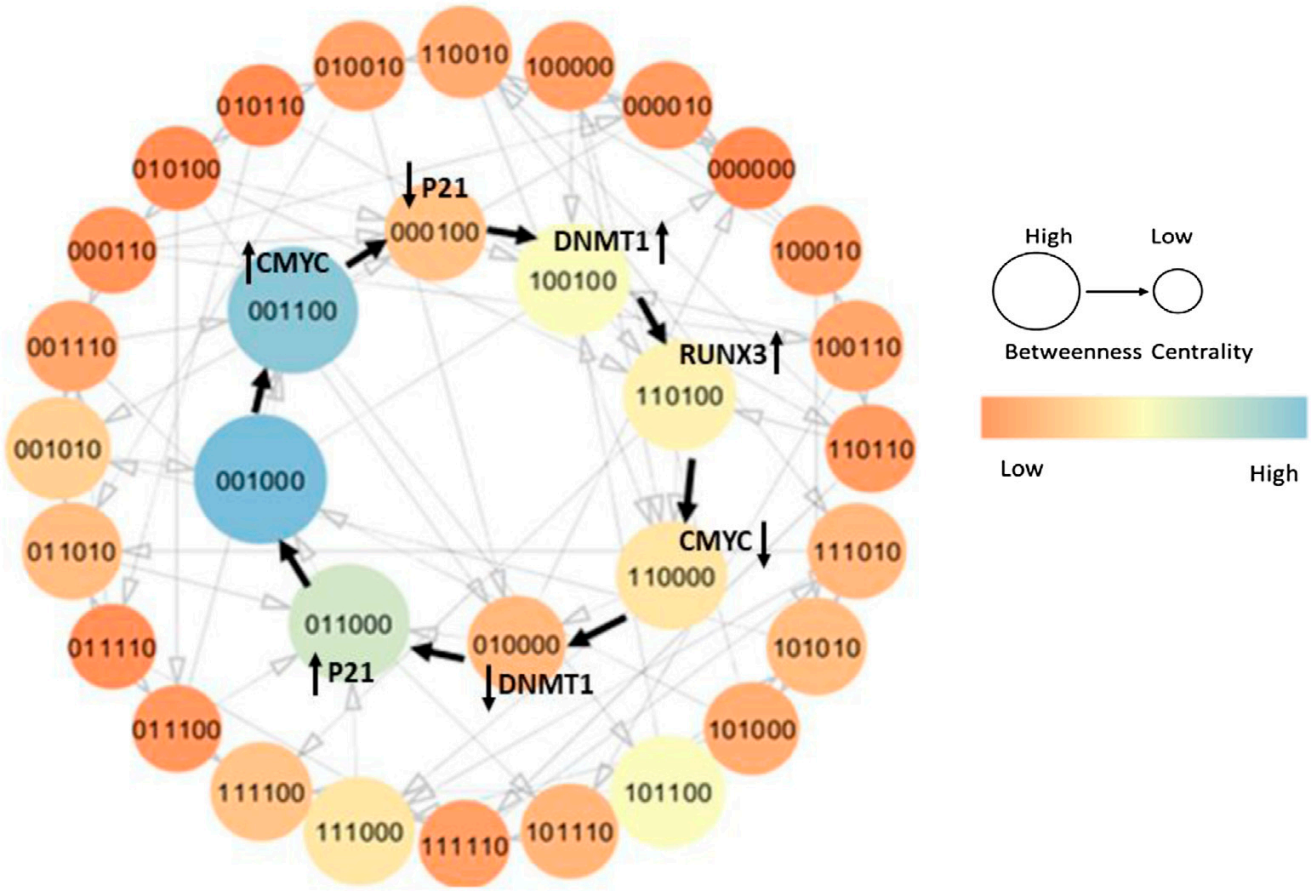
State graph of the recovery cycle from Model 6 (M6) is highlighted with black pointed arrows. Each circle indicates a unique qualitative state with gene entities in the following order: DNMT1, *RUNX3*, p21, c-myc, p53, and MDM2, sorted based on betweenness centrality. The recovery trajectory illustrates how a pathogenic system undergoes successive genetic evolution to reach normal homeostasis. The onset of oncogene c-myc introduces pathogenesis and tends to retain it by downregulating p21 and upregulating DNMT1. However, the activation of TSG *RUNX3* limits the overexpression of DNMT1 by inhibiting c-myc and restoring the p21 expression. The normal state characterized as (0,0,1,0,0,0) exhibits a high betweenness centrality as shown with a larger diameter and lighter color in the state graph. The color bar on the right side signifies the trend of betweenness centrality; that is, the lighter is the color and the larger is the diameter, the higher is the betweenness centrality of the qualitative state and vice versa.

**FIGURE 6:**
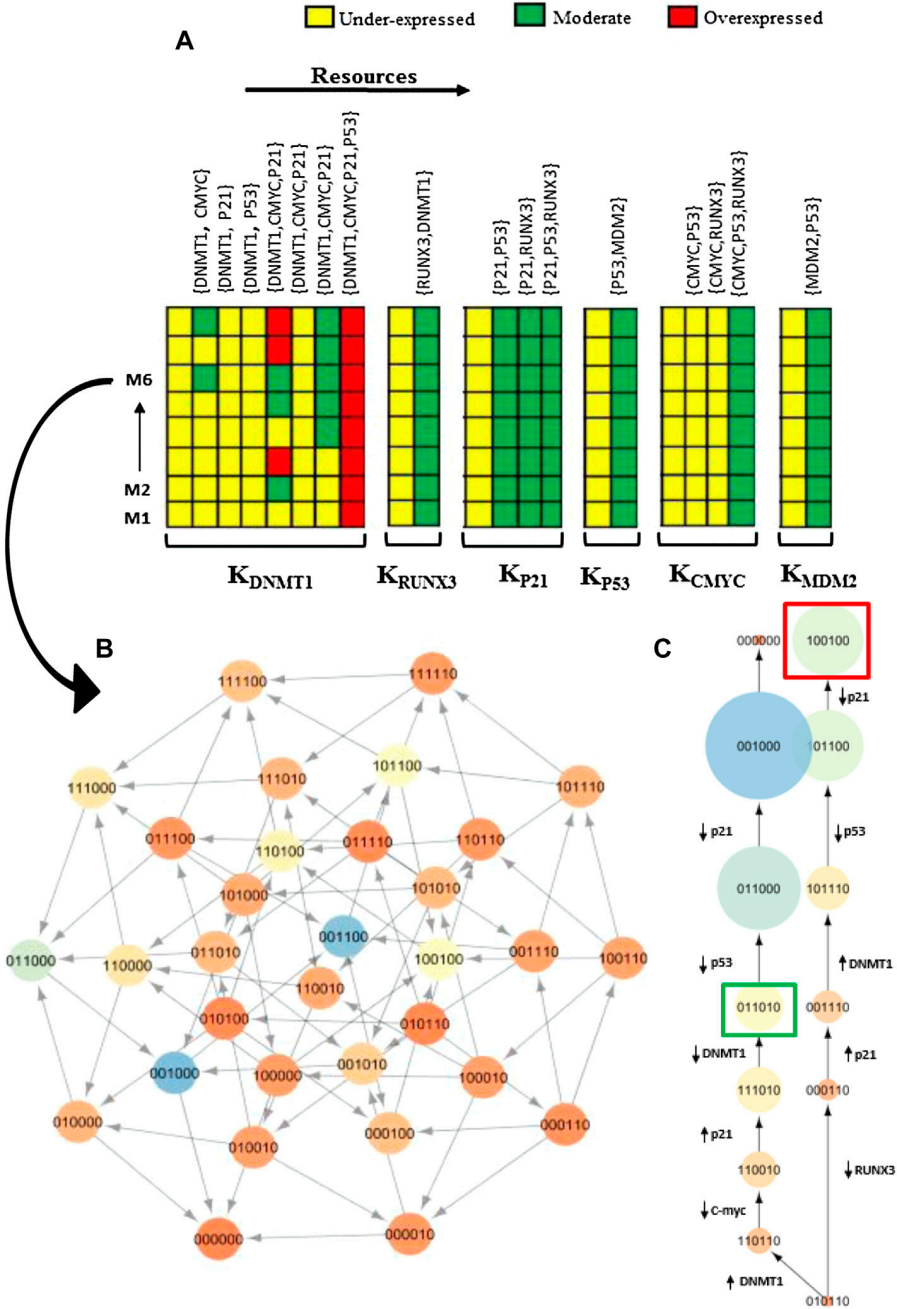
**(A)** Heat map of the first 8 sets of parameters out of total 14 (refer Figure 4) computed by SMBioNet is displayed, whereas Model 6 (M6) parameters were used to generate the state transition graph for network analysis. **(B)** State graph of M6 is shown, which composed of 32 nodes and 79 edges sorted on the basis of betweenness centrality. Each circle represents a unique state with gene expression in the order as follows: DNMT1, *RUNX3*, p21, c-myc, p53, and MDM2. The generated state transition graph illustrates all the possible qualitative states of the system. Trajectories of the graph were then further analyzed to identify important genetic evolution. **(C)** A bifurcation state is highlighted characterized by the qualitative state (010110). Trajectories display distinct paths from one common transition state characterized by the onset of oncogene c-myc leading to homeostasis or pathological loop based on successive genetic changes. From the bifurcating (0,1,0,1,1,0) state, the repressed *RUNX3* (0,0,0,1,1,0) converges the system toward pathological state with a successive onset of DNMT1 (1,0,1,1,1,0) and offset of p53 (1,0,0,1,0,0). Here, the qualitative state (1,0,0,1,0,0) is characterized as pathological state (highlighted in red box in the right trajectory) experienced by the system due to the consistent onset of oncogene c-myc along with persistent suppression of *RUNX3*. On the contrary, the system might evolve toward normal state (highlighted in green box in the left trajectory) if TSG *RUNX3* gets activated causing constant inhibition of oncogene c-myc (1,1,0,0,1,0), to control the moderate expression level of DNMT1 (0,1,1,0,1,0). The normal state is characterized by the activation of *RUNX3* along with the controlled expression level of DNMT1 (0,1,1,0,1,0), which is achieved by the system through continuous activation of TSG *RUNX3* along with the consistent inhibition of oncogene c-myc, ultimately leading to a typical reset state (0,0,0,0,0,0).

On the basis of selected logical parameters (M6) inferred from SMBioNet, a state graph was generated using GENOTECH and analyzed on the Cytoscape software to highlight significant genetic evolution including the recovery cycle (Figure 5). The state transition graph illustrates all the possible qualitative states exhibited by the DNMT1–*RUNX3* system as shown in Figure 6 (b). It contains a total of 32 nodes and 79 edges sorted on the basis of the betweenness centrality concept of graph theory. Our generated state graph highlights the temporal evolution of the system in the form of trajectories from one qualitative state to the other. It includes homeostasis, recovery trajectories, and bifurcation state from which the biological system can evolve either to homeostasis or to tumorigenesis. Here, we highlighted two important trajectories of the qualitative model involved in tumor progression and recovery/homeostasis. According to our model, the activation of oncogene c-myc signal (c-myc = 1) introduces pathogenesis in the biological system, which is characterized by the qualitative states (0,0,1,**1**,0,0) and (0,1,0,**1**,1,0) highlighted in Figures 5 and 6C, respectively.

### 3.3 Recovery

The qualitative model explains the step-by-step evolution of the system to recover from stress environment and maintain homeostasis. The onset of oncogenic c-myc (c-myc = 1) initiates the pathogenesis by inhibiting cell cycle arrest protein/TSG p21 (p21 = 0) leading to the qualitative state (0,0,0,1,0,0), which consequently activates DNMT1 (DNMT1 = 1) (1,0,0,1,0,0) in the system. It is a typical pathogenic state characterized by high expression level of DNMT1 and c-myc along with a suppressed concentration of TSGs, that is, *RUNX3*, p21, and p53 as shown in Figure 5.

DNMT1 activates *RUNX3* by maintaining the methylation status (*RUNX3* = 1) (1,1,0,1,0,0), which employs its tumor suppressor activity and inhibits the onset of oncogene c-myc subsequently, limiting the hyperactivation of DNMT1 leading to the states (1,1,0,0,0,0) and (0,1,0,0,0,0), respectively. In addition, *RUNX3* transactivates p21 (0,1,1,0,0,0) to further keep a concentration check of DNMT1 expression level. The restoration of p21 signal supervises the abnormal and uncontrolled proliferation, thus causing the system to recover from a pathogenic environment. These successive genetic changes evolve into homeostasis that is generally characterized by the presence of TSGs with a moderate concentration of DNMT1, and repressed oncogenes (0,0,1,0,0,0) as shown in Figure 5. Notably, the qualitative state (0,0,1,0,0,0) is the most central state of the system shown with lighter color and larger diameter. This suggests a high probability of the infected system to recover back to homeostasis through a series of steady states that are relatively less central (low betweenness centrality). The graph in Figure 5 with black pointed arrows describes all the states that the system exhibits to recover from a pathogenic state. Under normal circumstances, biological systems often exhibit oscillatory behavior or homeostasis during which the overall status of the system remains in a cycle of normal states. Thus, the qualitative model should expect pathogenic trajectories along with normal homeostasis behavior either as a cycle or as a closed path.

### 3.4 Bifurcation state

A transition bifurcation state highlighted in Figure 6 is characterized by the onset of oncogene c-myc from which the system could divert either toward pathogenic state or toward homeostasis depending on the successive genetic alterations. The suppressed level of TSG *RUNX3* (right trajectory of Figure 6C) converges the system toward pathological path in which the persistent activation of oncogene c-myc (c-myc = 1) with the subsequent consistent suppression of TSG *RUNX3* (*RUNX3* = 0) augments the hyperactivation of DNMT1. Consequently, the activated c-myc also downregulates p21 and p53 to further assist the metastasis and tumor invasion (1,0,0,1,0,0) characterized by red square in Figure 6C. Alternatively (left trajectory of Figure 6C), activation of TSG *RUNX3* inhibits the c-myc signaling and restores p21 expression, which controls the concentration of DNMT1 to maintain normal homeostasis of the system. This leads to normal qualitative state (0,1,1,0,1,0) characterized by the moderate level of DNMT1, the low expression of oncogenes, and the presence of TSGs along with the oscillations of the p53–MDM2 circuit as highlighted with green square on the left side of Figure 6C. Moreover, the qualitative normal state (0,0,1,0,0,0) in the homeostasis trajectory is well connected as shown with lighter color and larger diameter, which explains the probability of this state to occur more in the system than any other or pathogenic state (1,0,0,1,0,0).

### 3.5 Machine-Learning Models

ML models were built in WEKA using the DNMT1 data set of diverse structures and FDA-approved drugs (decitabine, azacytidine) that were extracted from the ChEMBL database (target ID ChEMBL1993). 80% of the collected DNMT1 data set was utilized for training the model, while the remaining 20% was included in the test set to validate the classification models. The number of training and test set compounds along with other details (bioactivities, activity threshold) is presented in Section 4, Supplementary Table S3. In order to build classification models, an activity threshold was established for both the training and test sets such that compounds with IC_50_ values ≤10 μM and >18 μM were categorized as active and least-active compounds, respectively. As a result, the training set contained 146 active (class label 1) and 68 least-active (class label 0) compounds. Initially, all the provided 2D MOE (2019.01) (ULC 2018) descriptors were calculated to train the DT model. The descriptors selected by the DT model were further utilized to build the ANN model of DNMT1 inhibitors using the MLP algorithm in WEKA. The descriptors including vsa_acc, h_logP, b_count, radius, PEOE_VSA-5, b_double, SlogP_VSA5, SMR_VSA2, Q_VSA_PNEG, and Kier3 (Wildman and Crippen 1999) were identified to be important for model learning and differentiating between active and least-active compounds efficiently. The detail of preferred descriptors is provided in Supplementary Table S4 of Section 4.

#### 3.5.1 Decision Tree

The J48 algorithm implemented in the ML software WEKA was used to build the DT classification model. Parameters of DT were optimized, and a pruned tree was built in order to get the best accuracy and performance on the available data set. Therefore, a minimum of 1 instance per leaf was set for the splitting rule and the rest default parameters of the J48 algorithm were utilized to build an optimal DT model. A final DT model with an overall size of 23 and 12 terminal nodes was obtained using the DNMT1 data set (shown in Section 4, Supplementary Figure S2). The overall topology of the DT classifier is elaborated in Section 4 of the Supplementary Material.

The model performance and overall predictive ability were evaluated through different statistical parameters including sensitivity (Eq. 2), specificity (Eq. 3), F-measure (Eq. 6), and MCC value (Eq. 7). The DT classifier attained accuracy, sensitivity, and specificity values of 0.974, 0.99, and 0.92, respectively, as shown in table 1. Overall, 97% of DNMT1 inhibitors were correctly classified into active and least-active class of compounds by the DT model. However, the ratios of correctly predicted active compounds (TPR) and the truly predicted least-active compounds (TNR) were observed to be 99% and 92%, respectively. The DT displayed an MCC value of 0.9 (table 1), indicating an optimal model efficiency with a strong positive correlation between the actual and predicted class labels. In addition, the F1-score was calculated to assess the balanced classification accuracy performance of the trained DT model, which turned out to be 0.98. As the DT showed an optimal performance on the training data in terms of classification and prediction, the descriptors identified by the DT model were further utilized to build the predictive ANN model.

**TABLE 1:** Statistical parameters of classification models, J-48 decision tree and MLP neural network, for training data calculated from WEKA.

ML algorithm	Accuracy	Sensitivity	Specificity	Precision	F-measure	MCC
Training set (80%)						
DT	0.974	0.993	0.920	0.973	0.983	0.932
ANN	0.969	0.979	0.940	0.979	0.979	0.919
Test set (20%)						
DT	0.826	0.916	0.500	0.868	0.891	0.45
ANN	0.717	0.805	0.400	0.828	0.816	0.20

#### 3.5.2 Artificial Neural Network

A set of 10 selected 2D descriptors [vsa_acc, h_logP, b_count, radius, PEOE_VSA-5, b_double, SlogP_VSA5, SMR_VSA2, Q_VSA_PNEG, and Kier3 (Wildman and Crippen 1999)] were used to build the ANN model in WEKA utilizing the MLP algorithm. Several ANN models were developed exploiting different combinations of parameters in order to obtain an optimal classification model. The final ANN comprised of 10 input nodes, 1 layer of 4 hidden nodes, and two output nodes (i.e., “1” for active and “0” for least-active compounds) (Section 4, Supplementary Figure S3). The ANN model was optimized using a training time of 500, learning rate of 0.3, and momentum of 0.2 to improve the accuracy and speed of learning.

The classification performance of ANN was also evaluated using statistical measures including classification accuracy, F1-score, and MCC values as reported in table 1. The trained ANN model attained a MCC value of 0.91, an overall classification accuracy of 0.97, and a F1-score of 0.98, thus indicating an optimal binary classification of our data set. Overall, 97% of DNMT1 data was correctly classified into active and least-active class by our learned ANN, whereby the true-positive and true-negative prediction rates were observed to be 0.98 and 0.94 implying a higher specificity and sensitivity, respectively. Both the trained DT and ANN classifiers displayed optimal statistical performance, that is, greater than 96% and a good predictive ability for the training data.

#### 3.5.3 Model Validation

The obtained DT and ANN models were validated using the 20% test set compounds that contain the most diverse chemical structures of the DNMT1 inhibitor data set (activity range of 0.01–132 μM). Overall, DT and ANN acquired a classification accuracy of greater than 70% for the test set whereby DT could correctly classify 83% of the data and the ANN model attained an accuracy of 73% (Table 1). Both the classifiers displayed an optimal value of sensitivity, that is, 0.92 and 0.80 for DT and ANN, respectively, implying a high true-positive prediction rate on the test set. Moreover, F1-score was calculated as 0.89 for the DT classifier and 0.81 for ANN indicative of the robustness and unbiased classification performance of our predictive ML classifiers. Despite the structural diversity of DNMT1 inhibitors, our models optimally retained the classification accuracy of 0.97 on the training set and 0.82 (DT) and 0.72 (ANN) on the test data. Notably, in comparison with the ANN model, the DT classifier could classify active and least-active compounds more efficiently as reported in table 1. The training and test sets utilized for model generation and validation in this study are shown in Section 2, Supplementary Tables S1, S2.

## 4 Discussion

TNBC is the most aggressive subtype of breast cancer that lacks obvious treatment options due to the availability of limited information about definite biomarkers. Previously, aberrant epigenetic modifications have been implicated in breast cancer, highlighting *RUNX3* as a promising prognostic biomarker (Wang et al., 2014). Moreover, several former studies have reported the depleted level of *RUNX3* in breast cancer cell lines predominantly due to local hypermethylation at the proximal promoter region of TSG *RUNX3,* which is an early event in carcinogenesis (Chen, 2012; Lau et al., 2006; Chen et al., 2016)*.* Therefore, the aberrant DNA methylation pattern is known as the hallmark of cancer epigenetics characterized by DNMT1, also known as the maintenance methyltransferase. So far, the individual role of *RUNX3* as a TSG in breast cancer and DNMT1 as the methylation maintenance protein has been reported in various scientific studies (Jiang et al., 2008; Chen et al., 2016; Lau et al., 2006; Subramaniam et al., 2009). However, the underlined epigenetic-mediated *RUNX3*-shared signaling that instigates and disseminates the tumorigenesis remains largely elusive. This study provides an insight into the integrated DNMT1–*RUNX3* signaling by taking into account various cancer-related significant upstream and downstream regulators (such as p21, c-myc, p53, and MDM2) and presents the DNMT1–*RUNX3* signaling cascades as one consolidated network. To the best of authors’ knowledge, this study is one of its kind to illustrate and model the epigenetic-mediated silencing of TSG *RUNX3* through the René Thomas framework modeling that has largely been a common practice of systems biology to investigate the dynamics of biological networks (Saeed et al., 2018).

Moreover, the model checking technique was adopted to build the interaction graph rendered as a state graph (Figure 6B) by utilizing the literature-driven information of the DNMT1–*RUNX3* signaling (Figure 2). The similar approach of the model checking has been previously applied in different former studies such as parameter estimation through formal modeling (Ahmad et al., 2012; Bibi et al., 2017), tail resorption in tadpole metamorphosis (Khalis et al., 2009), and immunity control in bacteriophage lambda (Richard, Comet, and Bernot 2006). We utilized this technique in the SMBioNet tool to exhaustively explore the model space of the DNMT1–*RUNX3* BRN for the estimation of precise parameters by encoding the existing wet-laboratory data in the form of computation tree logic (CTL) (Figure 3B). Once the computational verification with laboratory data is completed, the resultant model parameters and all its trajectories were further analyzed to understand how systematic evolution of the DNMT1–*RUNX3* system takes place with time, which might lead the system to invade cancer metastasis (Figure 6B).

As a result, two important behaviors of the DNMT1–*RUNX3* system were highlighted that shows the successive genetic events of the recovery cycle (Figure 5) and a bifurcation state leading to cancer invasion (Figure 6C). The trajectory (right side of Figure 6C) from our model plotted as a state graph articulates that the onset of oncogene c-myc infects the system and anticipates the upregulation of DNMT1 by downregulating the *RUNX3* expression level. It generates a feedback loop where the consistent onset of oncogene c-myc accompanied by the persistent suppression of TSG *RUNX3* contributes toward the overproduction of DNMT1. These findings are in agreement with the previous experimental studies, which reported the transcriptional upregulation of DNMT1 in TNBC due to the amplified c-myc expression level, emphasizing the importance of DNMT1 and c-myc relation in tumorigenesis (Wu et al., 2021).

On the contrary, the activation of *RUNX3* (left side of Figure 6C) and subsequent TSGs (such as p21, p53) regulate the adequate level of DNMT1 to acquire homeostasis. In addition, network analysis emphasized the tumor-suppressive role of *RUNX3* by demonstrating the higher probability of the infected system to recover to normal homeostasis in the presence of *RUNX3* (left side of Figure 6C) than its likelihood to invade the tumorigenesis. This is in line with the former findings that have conversely related the reactivation of *RUNX3* with a reduced potential of metastasis and invasiveness in breast cancer cells (Widschwendter and Jones, 2002). Although computational models cannot replace experiments, they are a step to demonstrate whether or not a proposed mechanism is sufficient to produce an observed phenomenon or an underlying assumption on the basis of their mathematical framework.

Moreover, the findings of our qualitative modeling suggested DNMT1 as a critical hub regulator of various oncogenic and tumor suppressor proteins that determine the ultimate status of the system. These outcomes advocate DNMT1 as a potential drug target in epigenetic cancer signaling through logic-based temporal evolution. Notably, the hypermethylation of TSGs by DNMT1 is a reversible process; therefore, *RUNX3* expression could be restored using demethylating compounds. Consequently, designing specific small modulators that inhibit DNMT1 activity in order to restore the TSG *RUNX3* could represent new clinical avenues for breast cancer therapeutics. Several pioneer studies have discussed the probable restoration of *RUNX3* and a reduced carcinogenic potential in cancer cell lines when treated with demethylating drugs (Lau et al., 2006; Jung, Park, Young Kim, et al., 2007).

To date, only two FDA-approved drugs (azacytidine and decitabine) are available that target DNMT1 in addition to other DNMTs for the treatment of myelodysplastic syndrome (Gnyszka, Jastrzębski, and Flis 2013). However, potential side effects of these drugs necessitate the design of less toxic and more specific inhibitors of DNMT1. Ever since the breakthrough of epigenetics in cancer treatment, several in silico studies such as pharmacophore and QSAR modeling (Yoo, Kim, and Robertson 2012; Phanus-Umporn et al., 2020) have been reported for drug discovery against DNMT1 through small modulators. However, to the best of authors’ knowledge, no model is developed that could learn the diverse features of DNMT1 inhibitors. Therefore, in this study, we adopted machine-learning approaches to identify potential 2D descriptors of DNMT1 modulators by utilizing all the available chemical scaffolds of DNMT1 inhibitors (i.e., natural compounds, synthetic, nucleoside inhibitors, and FDA drugs).

ML classifiers DT and ANN were developed to classify the active and least-active compounds of the DNMT1 data set. All 10 captured features of the best-performing model (DT and ANN) along with feature descriptions are provided in Section 4, Supplementary Table S4. The trained DT identified vsa_acc, h_logP, b_count, radius, PEOE_VSA-5, b_double, SlogP_VSA5, SMR_VSA2, Q_VSA_PNEG, and Kier3 as key descriptors of demethylating compounds targeting DNMT1. However, the overall vDW surface area of hydrogen bond acceptors (vsa_acc) was reflected as the most prominent and distinguishing 2D descriptor by the DT model. This feature has also been previously identified by Hassanzadeh et al. (2017), which further emphasizes the significance of HBA for the activity of DNMT1 modulators. Furthermore, the developed models were able to classify the training set and predict the test set with optimal accuracies (table 1). The final selected trained DT and ANN classifiers showed an optimal classification accuracy of 0.97 and high sensitivity and specificity along with MCC values of 0.93 and 0.92, respectively.

The subsequent screening of the diverse test set from the DT and the ANN model resulted in the classification accuracies of 0.83 and 0.72, respectively. However, the DT classifier outperformed the ANN model showing higher classification accuracy of 0.83 and sensitivity of 0.92. Notably, despite the structural diversity of the DNMT1 data set, the ML classifiers developed in the current study retained an optimal classification accuracy of 97% for the training data and greater than 70% for the test data. The relatively low predictive accuracy of ML models on the test set can be attributed to the diversity of DNMT1 inhibitors as they differ highly in terms of chemical structure, molecular weight, and other pharmacological variables such as clogP and lipophilicity. Therefore, the learned features of the training set might not be sufficient to fully predict and explain the behavior of the test set. Also, the FDA-approved drug (azacytidine) was fairly classified among most active modulators of DNMT1 by our developed model, which strengthens the classification and predictive ability of the ML models.

## 5 Conclusion

*RUNX3* has been proposed as a potential biomarker in TNBC for an early prognosis, which is known to be downregulated by DNMT1. However, the precise mechanism of epigenetic-mediated silencing of TSG *RUNX3,* which results in cancer invasion and metastasis, has not yet been explored. This study deals with the formal modeling of the DNMT1–*RUNX3* signaling and the development of ML models on a diverse data set of DNMT1 modulators. First, we employed a qualitative modeling approach to provide an insight into epigenetic-inspired *RUNX3* signaling. The results revealed that the onset of oncogene c-myc introduces pathogenesis in the system and its consistent activation along with persistent suppression of TSG *RUNX3* hyperactivates DNMT1 leading to cancer metastasis. Conversely, the activation of *RUNX3* leads the system to acquire normal homeostasis by transactivating other TSGs such as p21 and p53. Moreover, our findings advocate DNMT1 as a potential epigenetic drug target to revive the suppressed TSG *RUNX3* in breast cancer therapeutics. Second, predictive ML models (DT and ANN) have been developed that identify some potential 2D descriptors essential to modulate the DNMT1 activity, and the best-performing models have effectively classified the active and least-active inhibitors. The trained (DT and ANN) models acquired 97% classification accuracy on the training data set, and the subsequent screening of the test set through DT and ANN models achieved 83% and 72% predictive accuracy, respectively, emphasizing the optimal efficiency of the developed model. In general, the application of formal methods to unveil the network and model the underline genetic events responsible for DNMT1-inspired TSG *RUNX3* silencing along with ML approaches to predict the 2D attributes of hypomethylating compounds (targeting DNMT1) could present new computational avenues for the treatment of breast cancer requiring epigenetic therapy.

## Data Availability

The original contributions presented in the study are included in the article/Supplementary Material; further inquiries can be directed to the corresponding author.
