# Supplementary material for: Decoding the Role of Epigenetics in Breast Cancer Using Formal Modeling and Machine-Learning Methods
Source: Front Mol Biosci. 2022 Jul 11;9:882738. doi: 10.3389/fmolb.2022.882738 (PMC9309526; doi:10.3389/fmolb.2022.882738)
Supplement: Supplementary file 1 [file DataSheet1.docx]

Supplementary Material

Decoding the Role of Epigenetics in Breast Cancer Using Formal Modeling and Machine Learning Methods

Ayesha Asim, Yusra Sajid Kiani, Muhammad Tariq Saeed, Ishrat Jabeen^*^

School f Interdisciplinary Engineering and Sciences (SINES), National University of Sciences and Technology, NUST, Islamabad, Pakistan

* Correspondence:
Ishrat Jabeen
ishrat.jabeen@sines.nust.edu.pk

# Section 1: SMBioNet Code

**VAR**

DNMT1 = 0 2

RUNX3 = 0 1

P21 = 0 1

P53 = 0 1

CMYC = 0 1

MDM2 = 0 1

**REG**

P21 [(P21<1)]=> DNMT1

CMYC [(CMYC>=1)]=> DNMT1

P53 [(P53<1)]=> DNMT1 CMYC

DNMT1 [(DNMT1>=1)]=> RUNX3

RUNX3 [(RUNX3>=1)]=> P21

P53 [(P53>=1)]=> P21 MDM2

MDM2 [(MDM2<1)]=> P53

RUNX3 [(RUNX3<1)]=> CMYC

**PARA**

# Parameters for DNMT1

K_DNMT1 = 0

K_DNMT1+CMYC = 0 2

K_DNMT1+P21 = 0

K_DNMT1+P53 = 0

K_DNMT1+CMYC+P21 = 0 2

K_DNMT1+P21+P53 = 0

K_DNMT1+CMYC+P53 = 0 2

K_DNMT1+CMYC+P21+P53 = 2

# Parameters for RUNX3

K_RUNX3 = 0

K_RUNX3+DNMT1 = 1

# Parameters for P21

K_P21 = 0

K_P21+P53 = 1

K_P21+RUNX3 = 1

K_P21+P53+RUNX3 = 1

# Parameters for P53

K_P53 = 0

K_P53+MDM2 = 1

# Parameters for CMYC

K_CMYC = 0

K_CMYC+P53 = 0

K_CMYC+RUNX3 = 0

K_CMYC+P53+RUNX3 = 1

# Parameters for MDM2

K_MDM2 = 0

K_MDM2+P53 = 1

**CTL**

(EF(EG(DNMT1=2&RUNX3=0&CMYC=1&P21=0))

&

EF(EG(DNMT1=1&RUNX3=1&CMYC=0&P21=1)))

Section 2: Data Set of DNMT1

Supplementary Table 1: Canonical SMILE and CHEMBL IDs of training compounds along with their bioactivities are provided in the table below. The extreme right column shows class label of the compounds (1= active, 0= least actives).

| **CHEMBLID** | **SMILE** | **IC_50_μM** | **label** |
| --- | --- | --- | --- |
| CHEMBL140 | O=C(/C=C/c1cc(OC)c(O)cc1)CC(=O)/C=C/c1cc(OC)c(O)cc1 | 0.03 | 1 |
| CHEMBL3126646 | O=C(Nc1cc(Nc2nc(N)nc(C)c2)ccc1)c1cc(Nc2c3c(ncc2)cccc3)ccc1 | 0.12 | 1 |
| CHEMBL1201129 | O=C1N([C@@H]2O[C@H](CO)[C@@H](O)C2)C=NC(N)=N1 | 0.2 | 1 |
| CHEMBL2385821 | O=C(Nc1cc(Nc2nc(N)nc(C)c2)ccc1)c1ccc(Nc2c3c(ncc2)cccc3)cc1 | 0.37 | 1 |
| CHEMBL418052 | S(CC[C@H](N)C(=O)O)C[C@@H]1[C@@H](O)[C@@H](O)[C@H](n2c3ncnc(N)c3nc2)O1 | 0.8 | 1 |
| CHEMBL1328733 | S=C1N(c2ccccc2)C(=O)/C(=C\c2occc2)/C(=O)N1 | 0.81 | 1 |
| CHEMBL560106 | Clc1nc(N)c2ncn([C@H]3[C@H](O)[C@H](O)[C@@H](CS[C@H]4C[C@@H](C(=O)O)NC4)O3)c2n1 | 0.82 | 1 |
| CHEMBL2336409 | O=C(Nc1ccc(Nc2nc(N)nc(C)c2)cc1)c1ccc(Nc2c3c(ncc2)cccc3)cc1 | 0.85 | 1 |
| CHEMBL1303509 | Brc1cnc2n3C(=O)/C(=C/c4oc(-c5c(C(=O)O)cccc5)cc4)/Sc3nc2c1 | 1.02 | 1 |
| CHEMBL557902 | S(C[C@@H]1[C@@H](O)[C@@H](O)[C@H](n2c3ncnc(N)c3nc2)O1)[C@H]1C[C@@H](C(=O)O)NC1 | 1.1 | 1 |
| CHEMBL1373655 | O=C(O)c1cc(N2C(=O)/C(=C\C=C\c3occc3)/C(C)=N2)ccc1 | 1.18 | 1 |
| CHEMBL1704614 | O=C1C(=O)c2c(-c3c1c1c(cc3)cccc1)cccc2 | 1.34 | 1 |
| CHEMBL1464280 | O=C(Nc1cc(C2=NOC3(CN(C(=O)C4(C)CC4)[C@H](C(=O)N)C3)C2)ccc1)C(=C)C | 1.45 | 1 |
| CHEMBL552309 | S(CC[C@H](N)C(=O)O)C[C@@H]1[C@@H](O)[C@@H](O)[C@H](n2c3ncnc(N)c3cc2)O1 | 1.5 | 1 |
| CHEMBL1403497 | O=C1N(Cc2occc2)C(=O)CC(=O)N1 | 1.58 | 1 |
| CHEMBL318782 | ClC1=C(Cl)C(=O)c2c(C1=O)cccc2 | 1.6 | 1 |
| CHEMBL1890961 | O=C1C(=O)C=C(N2CC2)c2c1cccc2 | 1.87 | 1 |
| CHEMBL2349526 | Brc1c2c(N)ncnc2n([C@H]2[C@H](O)[C@H](O)[C@@H](CSCC[C@H](N)C(=O)O)O2)c1 | 1.9 | 1 |
| CHEMBL1579066 | Oc1c2c(O)cccc2cc2c1c(O)ccc2 | 1.9 | 1 |
| CHEMBL1360585 | O=C(Nc1cc(C2=NOC3(CN(C(=O)/C(=C\C)/C)[C@@H](C(=O)N)C3)C2)ccc1)C(=C)C | 1.94 | 1 |
| CHEMBL1360321 | O=[N+]([O-])c1oc(/C=C/2\C(=O)N(C)C(=O)NC\2=O)cc1 | 2.08 | 1 |
| CHEMBL1981446 | Fc1cc(Cn2c3c(c(/C=N\NC(=O)c4cc(O)cc(O)c4)c2)cccc3)ccc1 | 2.39 | 1 |
| CHEMBL559283 | Clc1nc(NCCc2ccc(c3ccccc3)cc2)c2ncn([C@H]3[C@H](O)[C@H](O)[C@@H](CS[C@H]4C[C@@H](C(=O)O)NC4)O3)c2n1 | 2.5 | 1 |
| CHEMBL538692 | S(CCC(N)C(=O)O)C[C@@H]1[C@@H](O)[C@@H](O)[C@H](n2c3nc(F)nc(N)c3nc2)O1 | 2.5 | 1 |
| CHEMBL1603637 | Clc1ccc(SCC=2/C(=C\c3ccc(OC)cc3)/C(=O)N(c3sc4c(n3)cccc4)N=2)cc1 | 2.86 | 1 |
| CHEMBL564768 | Clc1nc(N)c2ncn([C@H]3[C@H](O)[C@H](O)[C@@H](CSCCC(N)C(=O)O)O3)c2n1 | 2.9 | 1 |
| CHEMBL1566994 | S(=O)(=O)(Nc1ncnc(OC)c1)c1ccc(NC(=S)NC(=O)/C=C/c2occc2)cc1 | 2.92 | 1 |
| CHEMBL1543134 | O=C(NCC(=O)N=Nc1c(O)[nH]c2c1cccc2)/C=C/c1occc1 | 3.25 | 1 |
| CHEMBL1301673 | O=C(Nc1cc(C2=NOC3(CN(C(=O)/C=C/C=C/C)[C@@H](C(=O)N)C3)C2)ccc1)C(=C)C | 3.25 | 1 |
| CHEMBL1511981 | S(=O)(=O)(Nc1c(C(=O)O)cccc1)c1cc([N+](=O)[O-])c(N/N=C\c2ccc(SC)cc2)cc1 | 3.26 | 1 |
| CHEMBL1361920 | Clc1c(C(=O)O)cc(-n2c(C)c(/C=C\3/C(=O)N(Cc4ccccc4)C(=O)NC/3=O)cc2C)cc1 | 3.35 | 1 |
| CHEMBL1421172 | S=C1N(CCCC)C(=O)/C(=C/C=C/c2occc2)/C(=O)N1 | 3.39 | 1 |
| CHEMBL1995706 | Brc1cc(/C=N\Nc2c([N+](=O)[O-])cc(S(=O)(=O)Nc3c(C(=O)O)cccc3)cc2)c(OC)cc1 | 3.39 | 1 |
| CHEMBL1408747 | O=C(CN1C(=O)/C(=C/c2n(-c3ccc(C(=O)O)cc3)ccc2)/SC1=O)N1CCCC1 | 3.5 | 1 |
| CHEMBL540445 | O=C1C(=C)[C@H]2[C@H](O1)[C@H]1[C@](C)(O1)CC/C=C(/C)\CC2 | 3.5 | 1 |
| CHEMBL1423709 | S=C1N(Cc2ccc(F)cc2)C(=O)/C(=C/c2n(C)ccc2)/C(=O)N1 | 3.55 | 1 |
| CHEMBL1358848 | O=C(N1CCOCC1)C1=COC(=O)C=C1 | 3.67 | 1 |
| CHEMBL1501577 | Brc1ccc(S(=O)(=O)CCC(=O)Nc2c(C(=O)OC)cccc2)cc1 | 3.69 | 1 |
| CHEMBL555257 | S(C[C@@H]1[C@@H](O)[C@@H](O)[C@H](n2c3ncnc(NCCc4ccc(c5ccccc5)cc4)c3nc2)O1)[C@H]1C[C@@H](C(=O)O)NC1 | 3.7 | 1 |
| CHEMBL1373095 | S(=O)(=O)(NC1=NCCC1)c1cc(NC(=O)c2c3c(nc(-c4occc4)c2)cccc3)ccc1 | 3.71 | 1 |
| CHEMBL1561729 | O=C(O)c1ccc(-n2c(/C=C/3\C(=O)N(c4ccccc4)C(=O)N\3)ccc2)cc1 | 3.83 | 1 |
| CHEMBL1444349 | S=C1NC(=O)/C(=C\c2c([N+](=O)[O-])cc3OCOc3c2)/N1 | 3.85 | 1 |
| CHEMBL1981657 | O=[N+]([O-])N/C(=N\N=C\c1oc(-c2cc(C(=O)O)c(O)cc2)cc1)/N | 3.984 | 1 |
| CHEMBL1429479 | O=C/1N(c2ccc(C)cc2)C(=O)NC(=O)\C\1=C/c1cocc1 | 4 | 1 |
| CHEMBL551578 | S(C[C@@H]1[C@@H](O)[C@@H](O)[C@H](n2c3ncnc(N)c3nc2)O1)[C@H]1C[C@@H](C(=O)OC)NC1 | 4 | 1 |
| CHEMBL1303651 | Brc1ccc(-c2oc(/C=C\3/C(=O)N(C(C(=O)O)c4ccccc4)C(=S)S/3)cc2)cc1 | 4.08 | 1 |
| CHEMBL3126645 | O=C(Nc1ccc(Nc2nc(N)nc(C)c2)cc1)c1cc(Nc2c3c(ncc2)cccc3)ccc1 | 4.11 | 1 |
| CHEMBL1445488 | S(CC(=O)O)C=1C2=Nc3sc(C(=O)OCC)c(C)c3C(=O)N2C=C(C(=O)c2c(O)cccc2)C=1 | 4.12 | 1 |
| CHEMBL1485960 | S=C1N(c2cc(OCC)ccc2)C(=O)/C(=C/c2occc2)/C(=O)N1 | 4.24 | 1 |
| CHEMBL1558192 | Clc1c(-c2oc(/C=C\3/C(=O)N(C(C(=O)O)c4ccccc4)C(=S)S/3)cc2)cccc1 | 4.27 | 1 |
| CHEMBL1482590 | O=C(Nc1cc(C2=NOC3(CN(C(=O)CC)[C@@H](C(=O)N)C3)C2)ccc1)C(=C)C | 4.32 | 1 |
| CHEMBL1433062 | Clc1c(C2=NO[C@@H]3C(=O)N(c4c(OC)ccc(OC)c4)C(=O)[C@H]23)cccc1 | 4.35 | 1 |
| CHEMBL1412087 | Brc1ccc(-c2nc(N3C(=O)/C(=C/c4cc(OCC)c(O)cc4)/C(c4ccccc4)=N3)sc2)cc1 | 4.35 | 1 |
| CHEMBL2007178 | Brc1cc2c(oc(C(=O)N/N=C/c3n(-c4ccc(C(=O)O)cc4)ccc3)c2)cc1 | 4.36 | 1 |
| CHEMBL1397359 | O=[N+]([O-])c1sc(NC(=O)COC(=O)c2cc(OC)c(OC)cc2)nc1 | 4.45 | 1 |
| CHEMBL2005181 | O=C(O)c1c(-c2oc(/C=C\3/C(=O)n4c5c(c(C)cc(C)c5)nc4S/3)cc2)cccc1 | 4.45 | 1 |
| CHEMBL1439640 | O=C(Nc1sc(Cc2ccc(OC)cc2)nn1)Cc1sccc1 | 4.47 | 1 |
| CHEMBL551167 | S(C[C@@H]1[C@@H](O)[C@@H](O)[C@H](n2c3ncnc(N)c3nc2)O1)[C@@H]1CN(C)[C@H](C(=O)OC)C1 | 4.5 | 1 |
| CHEMBL1377441 | Clc1c(C(=O)O)ccc(-c2oc(/C=C\3/C(=O)N(Cc4ccc(Cl)cc4)C(=O)N/3)cc2)c1 | 4.54 | 1 |
| CHEMBL1967046 | c1(C2=Cn3c(nnc3)C=C2)occc1 | 4.58 | 1 |
| CHEMBL1430983 | Clc1ccc(CN2C(=O)/C(=C/c3cc(OC)c(O)c([N+](=O)[O-])c3)/SC2=O)cc1 | 4.6 | 1 |
| CHEMBL1346055 | Clc1cc(N2C(=O)/C(=C\c3n(-c4ccc(C(=O)O)cc4)ccc3)/NC2=O)ccc1 | 4.62 | 1 |
| CHEMBL1313324 | S=C1N(CCC(=O)O)C(=O)/C(=C/c2oc(-c3sc4c(n3)cccc4)cc2)/S1 | 4.66 | 1 |
| CHEMBL1985987 | O=C(Nc1ccc(/C(=N\NC(=O)c2cc(O)cc(O)c2)/C)cc1)Cc1ccccc1 | 4.69 | 1 |
| CHEMBL1566530 | Brc1cc(/C=C\2/C(=O)N(CC(=O)O)C(=S)S/2)c(OCc2c(Cl)cccc2)cc1 | 4.7 | 1 |
| CHEMBL564052 | S(C[C@@H]1[C@@H](O)[C@@H](O)[C@H](n2c3ncnc(N)c3nc2)O1)[C@H]1CNCC1 | 4.8 | 1 |
| CHEMBL1706577 | Fc1cc(/C=N\NC(=O)c2c3c(nc(-c4occc4)c2)cccc3)ccc1 | 4.81 | 1 |
| CHEMBL1411344 | S=C1NC(=O)/C(=C\c2n(-c3ccc(O)cc3)ccc2)/C(=O)N1 | 4.82 | 1 |
| CHEMBL1863716 | S(=O)(=O)(O)CCN1C(=O)/C(=C/c2c(-c3ccc(C)cc3)nn(-c3ccccc3)c2)/SC1=S | 4.93 | 1 |
| CHEMBL1702473 | O(C)C1=C(N)C(=O)c2nc(C)ccc2C1=O | 4.94 | 1 |
| CHEMBL1368108 | O=C(Nc1cc(C2=NOC3(CN(C(=O)C(=C)C)[C@H](C(=O)N)C3)C2)ccc1)C(=C)C | 4.95 | 1 |
| CHEMBL1310120 | Brc1oc(/C=C/2\C(=O)OC(C)=C\2)cc1 | 4.99 | 1 |
| CHEMBL1606403 | Clc1c(C(=O)OCC=C)cc(-c2oc(/C=C\3/C(C)=C(C#N)C(=O)NC/3=O)cc2)cc1 | 5 | 1 |
| CHEMBL560768 | Clc1nc(NCCc2ccc(c3ccccc3)cc2)c2ncn([C@H]3[C@H](O)[C@H](O)[C@@H](CS[C@@H]4CNCC4)O3)c2n1 | 5 | 1 |
| CHEMBL1536256 | S=C1N(CCC(=O)O)C(=O)/C(=C/c2c(-c3ccc(OC)cc3)nn(-c3ccccc3)c2)/S1 | 5.06 | 1 |
| CHEMBL1566980 | Brc1ccc(-c2nc(NC(=O)CSc3nc(O)c4c(n3)cccc4)sc2)cc1 | 5.12 | 1 |
| CHEMBL1490134 | S(=O)(=O)(Nc1c(C(=O)O)cccc1)c1cc([N+](=O)[O-])c(N/N=C\c2cc(OC)c(OCC=C)cc2)cc1 | 5.32 | 1 |
| CHEMBL1520133 | O=[N+]([O-])c1c(N(C)c2ccccc2)ccc(/C=C(/CCC(=O)O)\c2sc3c(n2)cccc3)c1 | 5.34 | 1 |
| CHEMBL1529471 | S=C1N(C(C(=O)Nc2cc(C(=O)O)c(O)cc2)C)C(=O)/C(=C/c2occc2)/S1 | 5.39 | 1 |
| CHEMBL599013 | O=C(O)c1c(/C=N\NC(=O)c2c3c(nc(-c4occc4)c2)cccc3)cccc1 | 5.4 | 1 |
| CHEMBL1978925 | Clc1c(C(=O)Oc2ccc(/C=N\NC(=O)c3cc(O)cc(O)c3)cc2)cccc1 | 5.4 | 1 |
| CHEMBL555352 | S(CCC(N)C(=O)O)C[C@@H]1[C@@H](O)[C@@H](O)[C@H](n2c3ncnc(NCCc4ccc(-c5ccccc5)cc4)c3nc2)O1 | 5.4 | 1 |
| CHEMBL1320181 | Brc1ccc(COc2c(OCC)cc(/C=C(\C#N)/C(=O)Nc3cc(C(=O)O)ccc3)cc2)cc1 | 5.4 | 1 |
| CHEMBL1414679 | Clc1ccc(C2C(C(=O)OC(C)C)=C(C)N=C3S/C(=C\c4ccc(OCC(=O)O)cc4)/C(=O)N23)cc1 | 5.43 | 1 |
| CHEMBL1509377 | Clc1ccc(OCC(=O)N2[C@@H](C(=O)N)CC3(ON=C(c4cc(NC(=O)C(=C)C)ccc4)C3)C2)cc1 | 5.52 | 1 |
| CHEMBL224857 | S=C1N(CCCCCC(=O)O)C(=O)/C(=C/c2c(-c3sccc3)nn(-c3ccccc3)c2)/S1 | 5.56 | 1 |
| CHEMBL1704267 | Fc1ccc(/C=N\NC(=O)c2c3c(nc(-c4occc4)c2)cccc3)cc1 | 5.57 | 1 |
| CHEMBL1440277 | S=C1N(CC(=O)O)C(=O)/C(=C/c2c(-c3ccc(OCC(C)C)cc3)nn(-c3ccccc3)c2)/S1 | 5.58 | 1 |
| CHEMBL1381264 | S(=O)(=O)(CC)C=1SC=2N(C(=N)/C(=C\c3oc(SCc4occc4)cc3)/C(=O)N=2)N=1 | 5.71 | 1 |
| CHEMBL1333249 | O=C(OCC(=O)C=1C(=O)N(C)C(=O)N(C)C=1N)c1c2c(nc(-c3occc3)c1)cccc2 | 5.78 | 1 |
| CHEMBL1485277 | S(=O)(=O)(Nc1c(C(=O)O)cccc1)c1cc([N+](=O)[O-])c(N/N=C\c2ccc(OCC)cc2)cc1 | 5.78 | 1 |
| CHEMBL1435948 | S(=O)(=O)(N1CCN(C(=O)C2Oc3c(OC2)cccc3)CC1)c1ccc(OC)cc1 | 5.82 | 1 |
| CHEMBL1340117 | O=C(Nc1cc(C2=NOC3(CN(C(=O)/C=C/CC)[C@@H](C(=O)N)C3)C2)ccc1)C(=C)C | 5.96 | 1 |
| CHEMBL560045 | S(CCC(N)C(=O)O)C[C@@H]1[C@@H](O)[C@@H](O)[C@H](n2c3nc(C)nc(N)c3nc2)O1 | 6 | 1 |
| CHEMBL1421559 | Clc1ccc(C(=O)c2c(C)oc-3c2C(=O)C(=O)c2c-3cccc2)cc1 | 6.05 | 1 |
| CHEMBL212414 | O=C(Nc1ccc(C)cc1)CN1C(=O)/C(=C\c2n(-c3cc(C(=O)O)ccc3)ccc2)/NC1=O | 6.05 | 1 |
| CHEMBL1317569 | O=C(N(CC(=O)NCc1occc1)C)COc1cc(C(=O)C)ccc1 | 6.09 | 1 |
| CHEMBL1369492 | S(=O)(=O)(Nc1sc(CC)nn1)c1ccc(NC(=S)NC(=O)/C=C/c2ccc(C(C)C)cc2)cc1 | 6.16 | 1 |
| CHEMBL1551448 | O=C(Nc1ccc(OC)cc1)CN(C(=O)c1c2c(nc(-c3occc3)c1)cccc2)C | 6.24 | 1 |
| CHEMBL1395998 | O=C(NCC(N(C)C)c1ccccc1)c1c2c(nc(-c3occc3)c1)cccc2 | 6.25 | 1 |
| CHEMBL1346592 | O=C(Nc1c(C)cccc1)CN1C(=O)/C(=C\c2n(-c3cc(C(=O)O)ccc3)ccc2)/NC1=O | 6.37 | 1 |
| CHEMBL1437464 | O=C(N1CC(C(=O)OCC)CCC1)c1c2c(nc(-c3occc3)c1)cccc2 | 6.42 | 1 |
| CHEMBL1391256 | O=C1C=2C(c3occc3)Nc3c(c4c(cc3)cccc4)C=2CCC1 | 6.62 | 1 |
| CHEMBL552763 | Clc1nc(NCCc2ccc(c3ccccc3)cc2)c2ncn([C@H]3[C@H](O)[C@H](O)[C@@H](CSCCC(N)C(=O)O)O3)c2n1 | 6.7 | 1 |
| CHEMBL1503050 | O=C(O)Cc1c2c(O)cccc2[nH]c1 | 6.74 | 1 |
| CHEMBL1443718 | O=C(OCC)C(C)N1C(=O)/C(=C/c2oc(-c3c(C(=O)O)cccc3)cc2)/SC1=O | 6.76 | 1 |
| CHEMBL104663 | S(=O)(=O)(Nc1ccc(Nc2c3c(nc4c2cccc4)cc([N+](=O)[O-])cc3)cc1)CCCNC(=N)N | 6.83 | 1 |
| CHEMBL1587837 | S(=O)(=O)(Nc1ccc(OC)cc1)c1cc(NC(=O)c2sc3n(-c4ccc(F)cc4)nc(C)c3c2)c(O)cc1 | 6.92 | 1 |
| CHEMBL1967028 | O=C(NCC(=O)N/N=C(/C)\c1ccc(CC)cc1)/C=C/c1occc1 | 6.95 | 1 |
| CHEMBL1505688 | S=C1N(CCCCCC(=O)O)C(=O)/C(=C\c2c(-c3sccc3)nn(-c3ccccc3)c2)/S1 | 6.97 | 1 |
| CHEMBL1560128 | O=C(OCC)c1n(-c2c(C(=O)O)cccc2)nc(-c2ccc(N3CCOCC3)cc2)c1 | 6.99 | 1 |
| CHEMBL1964442 | O=C(N/N=C\c1cocc1)c1cc(O)cc(O)c1 | 7.09 | 1 |
| CHEMBL1713628 | S(=O)(=O)(Nc1sccn1)c1cc(N=Nc2c([N+](=O)[O-])cccc2)c(N)cc1 | 7.09 | 1 |
| CHEMBL1361703 | O=C(NCc1c(OC)cccc1)COC(=O)c1c2c(nc(-c3occc3)c1)cccc2 | 7.11 | 1 |
| CHEMBL1347385 | S(c1oc(/C=C\2/C(=O)N(c3ccccc3)NC/2=O)cc1)c1c2c(ccc1)cccc2 | 7.12 | 1 |
| CHEMBL1509648 | O=[N+]([O-])c1c(NC(=O)/C=C/c2oc(C)cc2)cc(NC(=O)/C=C/c2oc(C)cc2)cc1 | 7.18 | 1 |
| CHEMBL617 | O=C(N[C@@H]1C(=O)N2C(C(=O)O)=C(COC(=O)C)CS[C@H]12)Cc1sccc1 | 7.28 | 1 |
| CHEMBL1315592 | O=C(N1CCC(C(=O)OC)CC1)c1c2c(nc(-c3occc3)c1)cccc2 | 7.31 | 1 |
| CHEMBL1434750 | Brc1cc(/C=C\2/C(=O)N(CC(=O)O)C(=S)S/2)c(OCc2ccc(Cl)cc2)cc1 | 7.36 | 1 |
| CHEMBL1569215 | Clc1ccc(N2C(=O)/C(=C/c3oc(-c4cc(C(=O)O)ccc4)cc3)/C(=O)N2)cc1 | 7.39 | 1 |
| CHEMBL560105 | S(CCC(N)C(=O)O)C[C@@H]1[C@@H](O)[C@@H](O)[C@H](n2c3ncnc(NCCCc4ccccc4)c3nc2)O1 | 7.4 | 1 |
| CHEMBL1320799 | S(=O)(=O)(N)c1ccc(N=Nc2c(O)nc(O)cc2)cc1 | 7.65 | 1 |
| CHEMBL1308055 | Clc1c(C)ccc(-n2c(/C=C/3\C(=O)N=C4SN=C(S(=O)(=O)C)N4C\3=N)ccc2)c1 | 7.72 | 1 |
| CHEMBL1312212 | S(=O)(=O)(Nc1cc(OC)ccc1)c1cc2c(NC(c3ccc(C(=O)O)cc3)C3C2C=CC3)cc1 | 7.94 | 1 |
| CHEMBL550440 | S(C[C@@H]1[C@@H](O)[C@@H](O)[C@H](n2c3ncnc(N)c3nc2)O1)[C@@H]1C[C@@H](C(=O)O)NCC1 | 8 | 1 |
| CHEMBL552246 | S(CCC(N)C(=O)O)C[C@@H]1[C@@H](O)[C@@H](O)[C@H](n2nnc3c(N)ncnc23)O1 | 8 | 1 |
| CHEMBL1893341 | O=C(Nc1c(C(=O)OC)ccc(C(=O)OC)c1)Cc1sccc1 | 8.17 | 1 |
| CHEMBL1715550 | O(C)c1c(O)ccc(/C=C/C=C/2\C(=O)/C(=C\C=C\c3cc(OC)c(O)cc3)/CC\2)c1 | 8.22 | 1 |
| CHEMBL1408320 | O=C1N(C(C)C)C(=O)/C(=C/C=C/c2occc2)/C(=O)N1Cc1occc1 | 8.31 | 1 |
| CHEMBL1571625 | S=C1NC(=O)/C(=C/C2C(=O)N(c3ccccc3)N=C2C)/N1 | 8.39 | 1 |
| CHEMBL1424189 | Clc1ccc(C2C(C(=O)OCC)=C(C)N=C3S/C(=C\c4ccc(OCC(=O)O)cc4)/C(=O)N23)cc1 | 8.42 | 1 |
| CHEMBL1983083 | O=[N+]([O-])c1ccc(NC(=O)COc2ccc(/C=N\NC(=O)c3ccc(O)cc3)cc2)cc1 | 8.43 | 1 |
| CHEMBL1423186 | S(CC(=O)Nc1c([N+](=O)[O-])cccc1)C1=NC(=O)CC(=O)N1 | 8.51 | 1 |
| CHEMBL1371301 | Brc1ccc(S(=O)(=O)CCC(=O)Nc2c(C)cc(N(CC)CC)cc2)cc1 | 8.64 | 1 |
| CHEMBL1522300 | O=C(N1CCN(C(=O)c2c3C(=O)N(c4ccc(OC)cc4)Cc3ccc2)CC1)c1occc1 | 9.04 | 1 |
| CHEMBL1558195 | S(=O)(=O)(N1CCOCC1)c1c(OCC)ccc(NC(=O)c2cc(-n3cccc3)ccc2)c1 | 9.08 | 1 |
| CHEMBL1521144 | Clc1ccc(C(=O)CSc2nc(O)c3c(n(-c4ccccc4)nc3)n2)cc1 | 9.12 | 1 |
| CHEMBL1480378 | S(=O)(=O)(Nc1nc(C)ccn1)c1ccc(NC(=O)Cc2sccc2)cc1 | 9.16 | 1 |
| CHEMBL256098 | O=C(Nc1c(C)cc(C)cc1)CN1C(=O)/C(=C/c2n(-c3cc(C(=O)O)ccc3)ccc2)/SC1=O | 9.17 | 1 |
| CHEMBL1181670 | N12c3c(-c4c(cccc4)C1=NCC2)cccc3 | 9.24 | 1 |
| CHEMBL1434555 | O=C(Nc1ccc(C)cc1)CN(C(=O)c1cc(-n2cccc2)ccc1)C | 9.26 | 1 |
| CHEMBL1464373 | S(=O)(=O)(Nc1ccc(C(=O)OCC(=O)NCc2occc2)cc1)c1sccc1 | 9.6 | 1 |
| CHEMBL1904582 | S(C(c1ccc(-c2ccccc2)cc1)(c1ccccc1)c1ccccc1)CC(N)C(=O)O | 9.65 | 1 |
| CHEMBL1605668 | S(CC(=O)O)Cc1c(OC)ccc(C(=O)/C=C/c2ccc(-c3ccccc3)cc2)c1 | 9.8 | 1 |
| CHEMBL1706407 | O=[N+]([O-])c1c(N(C)c2ccccc2)ccc(/C=C(\CCC(=O)O)/c2sc3c(n2)cccc3)c1 | 9.98 | 1 |
| CHEMBL1533351 | S=C1NC(=O)/C(=C\c2c(C)n(-c3c(C(=O)OCC)c4c(s3)CCCC4)c(C)c2)/C(=O)N1 | 10 | 1 |
| CHEMBL560505 | S(CCC(N)C(=O)O)C[C@@H]1[C@@H](O)[C@@H](O)[C@H](n2c3ncnc(NCc4cc(OC)cc(OC)c4)c3nc2)O1 | 18 | 0 |
| CHEMBL563938 | S(CCN)C[C@@H]1[C@@H](O)[C@@H](O)[C@H](n2c3ncnc(N)c3nc2)O1 | 18 | 0 |
| CHEMBL1507881 | O=C(O)c1c(C)c(-n2c(C)c(/C=C/3\C(=O)N(c4cc5OCOc5cc4)C(=O)NC\3=O)cc2C)ccc1 | 18 | 0 |
| CHEMBL1519992 | O=C(O)c1n(-c2c(C)cc(C)cc2)nc(-c2occc2)c1 | 18.1 | 0 |
| CHEMBL563782 | S(C[C@@H]1[C@@H](O)[C@@H](O)[C@H](n2c3ncnc(N)c3nc2)O1)C[C@H]1NCCC1 | 19 | 0 |
| CHEMBL1380580 | Fc1ccc(N=Nc2c(O)[nH]c3c2cccc3)cc1 | 19.3 | 0 |
| CHEMBL3414631 | O=C(O)[C@H](Cc1c2c([nH]c1)cccc2)N1C(=O)C=CC1=O | 20 | 0 |
| CHEMBL3109084 | S(N[C@H](C(=O)O)Cc1c2c([nH]c1)cccc2)c1c([N+](=O)[O-])cccn1 | 20 | 0 |
| CHEMBL560306 | S(CCC(N)C(=O)O)C[C@@H]1[C@@H](O)[C@@H](O)[C@H](n2c3ncnc(NCc4ccncc4)c3nc2)O1 | 22 | 0 |
| CHEMBL1508582 | O(C)c1c(OC)cc2-c3n(C(NCCCN(CC)CC)=Nc2c1)nc(-c1cnccc1)n3 | 23.4 | 0 |
| CHEMBL563024 | S(CCC(N)C(=O)O)C[C@@H]1[C@@H](O)[C@@H](O)[C@H](n2c3ncnc(NCCc4ccccc4)c3nc2)O1 | 27 | 0 |
| CHEMBL1446088 | Fc1c(NC(=O)CN(C(=O)c2cc(-n3cccc3)ccc2)CC)c(F)ccc1 | 27.4 | 0 |
| CHEMBL3126653 | O=C(Nc1ccc(N)cc1)c1ccc(Nc2c3c(ncc2)cccc3)cc1 | 27.9 | 0 |
| CHEMBL562689 | S(C[C@@H]1[C@@H](O)[C@@H](O)[C@H](n2c3ncnc(N)c3nc2)O1)C[C@@H]1NCCC1 | 31 | 0 |
| CHEMBL3126644 | O=C(Nc1c(Nc2nc(N)nc(C)c2)cccc1)c1ccc(Nc2c3c(ncc2)cccc3)cc1 | 35 | 0 |
| CHEMBL538627 | Clc1nc(N)c2ncn([C@H]3[C@H](O)[C@H](O)[C@@H](CS[C@@H]4CNCC4)O3)c2n1 | 36 | 0 |
| CHEMBL3126649 | O=C(Nc1cc(Nc2nc(N)nc(C)c2)ccc1)c1c(Nc2c3c(ncc2)cccc3)cccc1 | 38.6 | 0 |
| CHEMBL3109085 | S(N[C@H](C(=O)O)Cc1c2c([nH]c1)cccc2)c1c([N+](=O)[O-])cc([N+](=O)[O-])cc1 | 40 | 0 |
| CHEMBL1607517 | O=C1c2c3c(ccc2)cccc3-c2c1cccc2 | 41 | 0 |
| CHEMBL1441143 | O=C(Nc1sc(CCCCCCCC)nn1)c1occc1 | 42.4 | 0 |
| CHEMBL551100 | S(C[C@@H]1[C@@H](O)[C@@H](O)[C@H](n2c3ncnc(N)c3nc2)O1)[C@@H]1CNCC1 | 44 | 0 |
| CHEMBL561047 | S(CCC(N)C(=O)O)C[C@@H]1[C@@H](O)[C@@H](O)[C@H](n2c3c(c(N)ncc3)nc2)O1 | 45 | 0 |
| CHEMBL563946 | S(CCC(N)C(=O)O)C[C@@H]1[C@@H](O)[C@@H](O)[C@H](n2c3nccc(N)c3nc2)O1 | 45 | 0 |
| CHEMBL383475 | O=C(O)C(Cc1c2c([nH]c1)cccc2)N1C(=O)c2c(C1=O)cccc2 | 50 | 0 |
| CHEMBL3109076 | O=C(O)[C@@H]1N(C(=O)c2cc3c(cc2)cccc3)CC[C@@H]1Cc1c2c([nH]c1)cccc2 | 50 | 0 |
| CHEMBL556265 | S(CCC(N)C(=O)O)C[C@@H]1[C@@H](O)[C@@H](O)[C@H](n2c3ncnc(NCc4ccccc4)c3nc2)O1 | 61 | 0 |
| CHEMBL1472200 | O=C(OCC)Cc1nc(NC(=O)c2c(N)c3c(s2)nc2c(c3)ccc(C)c2)sc1 | 70.9 | 0 |
| CHEMBL3109078 | O=C(O)[C@@H]1N(C(=O)c2ccccc2)CC[C@@H]1Cc1c2c([nH]c1)cccc2 | 73 | 0 |
| CHEMBL560165 | S(CCC(N)C(=O)O)C[C@@H]1[C@@H](O)[C@@H](O)[C@H](n2c3nc(OC)nc(N)c3nc2)O1 | 75 | 0 |
| CHEMBL115145 | O=C(O)c1c(O)ccc(Cc2cc(C(=O)O)c(O)cc2)c1 | 92 | 0 |
| CHEMBL559281 | S(C[C@@H]1[C@@H](O)[C@@H](O)[C@H](n2c3ncnc(N)c3nc2)O1)[C@H]1[C@@H](N)CCC1 | 94 | 0 |
| CHEMBL3109077 | O=C(O)[C@H]1N(C(=O)c2ccccc2)CC[C@H]1Cc1c2c([nH]c1)cccc2 | 98 | 0 |
| CHEMBL3109075 | O=C(O)[C@H]1N(C(=O)c2cc3c(cc2)cccc3)CC[C@H]1Cc1c2c([nH]c1)cccc2 | 128 | 0 |
| CHEMBL1916517 | O=[N+]([O-])c1ccc(C2=NOC(CN(C)C)C2)cc1 | 150 | 0 |
| CHEMBL403716 | O=[N+]([O-])c1ccc(C=2O[C@@H](CN3CCCC3)CN=2)cc1 | 150 | 0 |
| CHEMBL549412 | S(CCC(N)C(=O)O)C[C@@H]1[C@@H](O)[C@@H](O)[C@H](n2c3ncnc(Nc4ccccc4)c3nc2)O1 | 154 | 0 |
| CHEMBL3126654 | O=C(Nc1ccc(Nc2nc(N)nc(C)c2)cc1)c1ccc(N)cc1 | 173 | 0 |
| CHEMBL3126647 | O=C(Nc1c(Nc2nc(N)nc(C)c2)cccc1)c1cc(Nc2c3c(ncc2)cccc3)ccc1 | 174 | 0 |
| CHEMBL3263626 | O[C@H]1[C@@H](O)[C@H](n2c3ncnc(N)c3nc2)O[C@@H]1CC(N)CC1CCCCC1 | 179 | 0 |
| CHEMBL597113 | Brc1cc(C(=O)c2ccc(OCC(=O)O)cc2)c(OCC(=O)O)cc1 | 212 | 0 |
| CHEMBL558406 | S(CCC(N)C(=O)O)C[C@@H]1[C@@H](O)[C@@H](O)[C@H](n2c3nc(SC)nc(N)c3nc2)O1 | 216 | 0 |
| CHEMBL3109080 | O=C(O)[C@H](Cc1c2c(sc1)cccc2)N1C(=O)c2c(C1=O)cccc2 | 230 | 0 |
| CHEMBL1916669 | N(CC1ON=C(c2ccc(N)cc2)C1)(C)C | 270 | 0 |
| CHEMBL563570 | S(CC[C@H](N)C(=O)O)C[C@@H]1[C@@H](O)[C@@H](O)[C@H](n2c3ncncc3nc2)O1 | 300 | 0 |
| CHEMBL1235825 | O=C(O)[C@@H](N)CCN(C[C@@H]1[C@@H](O)[C@@H](O)[C@H](n2c3ncnc(N)c3nc2)O1)C | 302 | 0 |
| CHEMBL1916683 | O=[N+]([O-])c1ccc(CC2=NOC3C2CN(C)C3)cc1 | 310 | 0 |
| CHEMBL1564869 | O=C(O)[C@H](Cc1c2c([nH]c1)cccc2)N1C(=O)c2c(C1=O)cccc2 | 390 | 0 |
| CHEMBL1916680 | Nc1ccc(C2=NOC3C2CN(C)C3)cc1 | 570 | 0 |
| CHEMBL1916684 | O(C)c1ccc(CC2=NOC3C2CN(C)C3)cc1 | 1130 | 0 |
| CHEMBL1916672 | O=[N+]([O-])c1ccc(CC2=NOC(CN(C)C)C2)cc1 | 1600 | 0 |

Supplementary Table 2: SMILE representation of test data with CHEMBL reference ID, IC_50_ values and respective class label is provided here.

| **CHEMBLID** | **SMILE** | **IC_50_μM** | **label** |
| --- | --- | --- | --- |
| CHEMBL83747 | Brc1c(O)ccc(C/C(=N/O)/C(=O)NCCSSCCNC(=O)/C(=N/O)/Cc2cc(Br)c(O)cc2)c1 | 0.018 | 1 |
| CHEMBL297453 | O(CCCC[N+](CC)(CC)C)c1cc2C(=O)c3c(-c2cc1)ccc(OCCCC[N+](CC)(CC)C)c3.[I-].[I-] | 0.5 | 1 |
| CHEMBL3126651 | Cl/N=C\1/C(=N/Cl)/C=CC=C/1 | 0.65 | 1 |
| CHEMBL275938 | S(=O)(=O)([O-])CC(NC(=O)CC[N+](CCO)(C)C)(C)C | 0.68 | 1 |
| CHEMBL1988862 | O=C(O)/C(=C\CCC(C)[C@@H]1[C@]2(C)[C@](C)(C3=C([C@]4(C)[C@H](C(C)(C)C(=O)CC4)CC3)CC2)CC1)/C | 1.02 | 1 |
| CHEMBL1789994 | O=C(Nc1c2C(=O)c3c(C(=O)c2ccc1)cccc3)CCN(CC)CC.Cl | 1.24 | 1 |
| CHEMBL1321933 | O=C(O[C@H]1[C@@H](c2cc(O)c(O)c(O)c2)Oc2c(c(O)cc(O)c2)C1)c1cc(O)c(O)c(O)c1 | 1.29 | 1 |
| CHEMBL3126652 | O=C(O)/C=C/C(=O)O.O(C)c1c(Oc2ccc(OC)cc2)c2c(c(NCc3ccc(CN)cc3)c1)nccc2 | 2.23 | 1 |
| CHEMBL1539325 | O=[N+]([O-])C1=C(O)C(=O)C=C2C1=NC1C32C2N(CC4=CCOC(CC(=O)OC)C1C4C2)CC3 | 2.92 | 1 |
| CHEMBL1464200 | O=CC(O)C1C(O)C(O)C(=O)O1 | 3.2 | 1 |
| CHEMBL1469245 | O=C(OC)/C(=C(/N(C)C)\C=C\C=C\C=C\N(C)C)/C(=O)C | 3.35 | 1 |
| CHEMBL1729254 | O=C(Nc1ccc(Nc2c3c(ncc2)cccc3)cc1)c1ccc(Nc2c3c(ncc2)cccc3)cc1 | 3.5 | 1 |
| CHEMBL1533230 | O=C(NCCN(CC)CC)c1ccc(NCCCCCCCCCCCCN2C(=O)c3c(C2=O)cccc3)cc1 | 3.6 | 1 |
| CHEMBL1510676 | S(=O)(=O)(N)c1ccc(NC(=O)COC(=O)c2sc([N+](=O)[O-])cc2)cc1 | 3.74 | 1 |
| CHEMBL1315784 | Clc1c(Cl)cc(Cl)c2NC(C(=O)O)C3C(c12)C=CC3 | 3.81 | 1 |
| CHEMBL3414630 | O=C(O)[C@@H](N)CCN(CCc1c2c(sc1)cccc2)C[C@@H]1[C@@H](O)[C@@H](O)[C@H](n2c3ncnc(N)c3nc2)O1 | 4 | 1 |
| CHEMBL1972915 | Fc1c(NC(=O)CN2C(=O)c3c(C)n(Cc4c(F)cccc4)c(C)c3C=N2)c(F)c(F)cc1F | 4.29 | 1 |
| CHEMBL1456035 | C(Cc1n2c(nn1)C=C(C)C=C2)c1n2c(nn1)C=C(C)C=C2 | 4.36 | 1 |
| CHEMBL1489 | Clc1ccc(N2C(=O)/C(=C\c3c(C)n(-c4c(C#N)c5c(s4)CCCC5)c(C)c3)/C(=O)NC2=O)cc1 | 5 | 1 |
| CHEMBL1523953 | O=C1C(c2ccc(O)cc2)=COc2c1c(O)cc(O)c2 | 5.04 | 1 |
| CHEMBL1440076 | Brc1ccc(/C=C/C(=O)NC(=S)Nc2ccc(S(=O)(=O)Nc3sccn3)cc2)cc1 | 5.22 | 1 |
| CHEMBL1389315 | O=C(/C=C/C=C/C)[C@@H]1[C@@H]([C@@]2(C)C(=O)C(C)=C(O)O2)[C@@H]2C(O)(C)C(=O)[C@@]1(C)C(O)=C2C(=O)/C=C/C=C/C | 5.23 | 1 |
| CHEMBL1322547 | BrC=1C(=O)OC=C(C(=O)N2CC(C)(C)CCC2)C=1 | 5.25 | 1 |
| CHEMBL1568915 | O=C(O)c1c(O)c(C/C=C(\CC/C=C(\C)/C)/C)c(OC)cc1CCc1ccccc1 | 5.93 | 1 |
| CHEMBL1975589 | S(C[C@@H]1[C@@H](O)[C@@H](O)[C@H](n2c3ncnc(N)c3nc2)O1)C1CNC1 | 6.06 | 1 |
| CHEMBL1571682 | O=C(NCC=1C(=O)NC(C)=CC=1CCC)c1c2c(n(C(C)C)nc2)cc(-c2cnc(N3CCN(C)CC3)cc2)c1 | 6.86 | 1 |
| CHEMBL1902527 | BrC1=C(OC)C(=O)c2c(C1=O)nc(C)cc2 | 7.2 | 1 |
| CHEMBL1209491 | O=C(O)c1c(O)ccc(/C(/c2cc(C(=O)O)c(O)cc2)=C\2/C=C(C(=O)O)C(=O)C=C/2)c1 | 7.56 | 1 |
| CHEMBL1438370 | S(=O)(=O)(O)c1cc([N+](=O)[O-])c(O)c(N)c1 | 7.6 | 1 |
| CHEMBL1461389 | Clc1n(-c2ccccc2)nc(C)c1/C=N\Nc1c([N+](=O)[O-])cc(S(=O)(=O)Nc2c(C(=O)O)cccc2)cc1 | 7.7 | 1 |
| CHEMBL1897695 | S(=O)(=O)(Nc1n(C2CCCCC2)c2nc3c(nc2c1S(=O)(=O)c1ccccc1)cccc3)c1ccccc1 | 8.25 | 1 |
| CHEMBL1348946 | O=C1c2sccc2-c2c3c(cccc13)ccc2 | 8.28 | 1 |
| CHEMBL1548829 | Clc1c(Cl)ccc(NC(=O)Nc2sc(-c3ccncc3)nn2)c1 | 8.38 | 1 |
| CHEMBL1341590 | O=C1N=C(NCc2occc2)NCN1 | 8.67 | 1 |
| CHEMBL1707222 | Brc1cc2sc(NC(=O)CSc3c(C#N)c4nc(O)cc(O)c4s3)nc2cc1 | 8.73 | 1 |
| CHEMBL1612983 | S(N1[C@H](C(=O)O)[C@H](Cc2c3c([nH]c2)cccc3)CC1)c1c([N+](=O)[O-])cc([N+](=O)[O-])cc1 | 9.52 | 1 |
| CHEMBL3414632 | O=C1/C(=C/C=2C(=O)c3c(OC=2)cccc3)/C(=N)N2N=C(c3occc3)SC2=N1 | 19 | 0 |
| CHEMBL558882 | S(=O)(=O)(Nc1c(N2CCCC2)ccc(C(F)(F)F)c1)c1ccc(CCC(=O)O)cc1 | 21 | 0 |
| CHEMBL2063048 | O=C(Nc1ccc(Nc2nc(N)nc(C)c2)cc1)c1ccc(Nc2nc(N)nc(C)c2)cc1 | 22 | 0 |
| CHEMBL1458444 | O(C)c1c(O[C@H]2[C@H](O)[C@@H](O)[C@H](O)[C@@H](CO)O2)cc2OC(=O)C(Oc3cc4OC(=O)C=Cc4cc3)=Cc2c1 | 25.1 | 0 |
| CHEMBL44 | S(CC(=O)Nc1c(C)cccc1)c1c([N+](=O)[O-])scc1 | 30 | 0 |
| CHEMBL539449 | Clc1c2NC(=O)C(=O)c2cc(Cl)c1 | 44 | 0 |
| CHEMBL3109079 | O=C(Nc1c(C(=O)OC)c2c(s1)CCCCC2)COC(=O)c1ccc(NC(=O)CC#N)cc1 | 80 | 0 |
| CHEMBL382617 | O=C1N([C@H]2[C@H](O)[C@H](O)[C@@H](CO)O2)C=NC(N)=N1 | 100 | 0 |
| CHEMBL2204997 | S(=O)(=O)(N1[C@@H](c2ccc(C(C)(C)C)cc2)C(C(=O)O)=CC[C@H]1c1ccc(C(C)C)cc1)c1ccc(C)cc1 | 121 | 0 |
| CHEMBL2018855 | S(=O)(=O)(Nc1ccc(C(=O)O)cc1)c1cc2c(NC(c3c(F)cccc3)C3C2C=CC3)cc1 | 132 | 0 |

Supplementary Figure 1: The data of DNMT1 inhibitors exhibit an activity range of IC_50_ 0.01-1600 µM where majority of the inhibitors (almost 45%) display an IC_50_ value less than 5.5 µM therefore a threshold of IC_50_ 10 µM (pIC_50_ = 5) was defined for active (1) and least active inhibitors (0). To plot the data binarization, IC_50_ values are converted into pIC_50_ (-log of IC_50_) such that IC_50_ 1 µM = 1 x10^-6 M pIC_50_.

# Section 3: DNMT1-*RUNX3* integrated signaling network

DNMT1 along with other regulators (UHRF1, HAUSP, Tip60, HDAC1 and PCNA) is recruited on the replication fork during S-phase, to form a macro-protein complex. In a series of events at the replication fork at first, the UHRF1 recognizes the hemi-methylated DNA and facilitates DNMT1 at the replication fork to methylate the newly synthesized DNA strand. DNMT1 in complex with UHRF1, then transfers the methyl group on the daughter strand via flip mechanism as demonstrated by (J. Yoo et al. 2012). Moreover, during methylation activity the UHRF1 tends to proteasomaly degrade DNMT1 because of its native E3 ligase activity domain. However, the regulators including HAUSP (herpesvirus-associated ubiquitin-specific protease) (figure 2) and HDAC1 (Histone Deacetylase 1), counteract the E3 ligase activity of UHRF1 and inhibits the acetylation activity of Tip60 enzyme respectively, to ensure the presence of DNMT1 at the replication fork for successful methylation of daughter strand. Notably, the concerted actions of DNMT1 protectors (HAUSP and HDAC1) and functional destroyers (Tip60 and UHRF1) in the macro-protein complex maintain the adequate concentration of DNMT1 at different stages of cell cycle. As a result, daughter strand acquires the normal status of methylation with a hypomethylated promoter region and subsequently translated into a tumor suppressor protein in various cancer signaling pathways such as TGF-β, Wnt/β-catenin and Mitogen K-Ras.

The **TGF-β (**Transforming Growth Factor beta) cancer signaling pathway is well-known to suppress tumorigenesis mainly by inducing the programmed cell death or inhibiting the cell growth and proliferation (Y and P 2011). However, an impairment in the TGF-β signaling pathway is evident due to the loss of function or deficient TSG *RUNX3* in the cell (Han et al. 2003). Generally, upon stimulation by the exclusively expressed cytokine, the TGF-β receptor is activated in the cell. Subsequently, the *RUNX3* directly binds with signal transducers SMADs (R-smad and smad 4) to upregulate the transcription of target genes Bim and p21, inducing programmed cell death and cell growth inhibition (Chuang and Ito 2010b), respectively (figure 2). Moreover, *RUNX3* inhibits the process of angiogenesis by inhibiting the activation of major angiogenic factor VEGF to limit the cancer cell invasion and metastasis (F. Chen et al. 2014).

Likewise, **Wnt/β-catenin** signaling is critical for cancer cell invasion, endothelial mesenchymal transition (EMT), cell proliferation and cell growth in tumor development. The activation of **Wnt/β-catenin** is often reported in several human carcinomas including breast cancer and hepatocellular cancer (H, E, and T 2011). In practice, oncogenic **Wnt** signaling is sustained through the accumulation of β -catenin concentration in the cytoplasm. It is notable that a high concentration of β-catenin changes the expression of target genes by binding TCF4 transcription factors. However, *RUNX3* attenuates the Transcription Factor 4 (TCF4) β-catenin complex consequently, preventing the transcriptional activation of target oncogenes including c-myc and cyclin D1 (Ito 2011) (figure2). In addition, *RUNX3* is known to suppress the TGF-beta and **Wnt** mediated EMT as shown in figure 2; a process whereby epithelial cells undergo a shift and acquire the ability to disseminate, invade and cause metastasis (Chuang and Ito 2010b; Lau et al. 2006).

**Mitogen K-Ras** is another entity that is frequently mutated in human carcinomas including lung adenocarcinomas (ADCs). The oncogenic activation of K-Ras induces premature senescence early in tumorigenesis in the presence of TSGs, through the activation of cellular defense against the oncogenic insult (YS et al. 2020). This tumor suppressive effect is mediated by the transcription factor *RUNX3*, which maintains its binding with bromodomain-containing 2 (BRD2) and p300 signal transducers thereby, facilitating the transcription of ARF and p21 (Lee et al. 2013) (figure 2). Subsequently, ARF induces p53 dependent cell apoptosis and p21 causes cell cycle arrest in response to the oncogenic Ras. Moreover, p21 is induced in both p53 dependent and independent manner which plays a key role in the cellular defense against environmental stress (F. Chen et al. 2016).

In the late S phase, when DNA replication and methylation is completed and there is no requirement of DNMT1 in the cell, the cellular changes such as non-availability of hemi methylated DNA and other histone modifications trigger the ubiquitination-mediated proteasomal degradation of DNMT1 (Bronner 2011; Du et al. 2010) (figure 2). The concentration of DNMT1 is tracked in different stages of cell cycle alongside the process of DNA replication. Whereby, the concentration of DNMT1 remains highest in the S phase, lowers after S phase and turns out to be the lowest during G1 phase of the cell cycle (Robertson et al. 2000; Szyf, Bozovic, and Tanigawa 1991; Du et al. 2010)

# Section 4: Machine Learning Results

**Decision Tree:** The trained DT identified vsa_acc, h_logP, b_count, radius, PEOE_VSA-5, b_double, SlogP_VSA5, SMR_VSA2, Q_VSA_PNEG and Kier3 as key descriptors of DNMT1 inhibitors. The topology of DT presents vsa_acc as the root node and the key decisive descriptor for the data set of DNMT1 inhibitors. The vsa_acc node holds highest normalized information of the data that is further divided on the basis of various internal or terminal nodes (2D descriptors including h_logP and b_count etc). Each path from root to leaf represents a distinct rule that collectively makes a classification tree. For instance, the compounds displaying vsa_acc values >32.40 were categorized as active DNMT1 inhibitors. Additionally, the compounds having vsa_acc ≤ 32.4, h_logP ≤ 4.56, b_count >29 and radius >10 were categorized as the active class of compounds (represented as 1). However, for radius values ≤ 10 and PEOE_VSA-5 values >33.78 the compounds were classified as least active inhibitors (labeled as 0) of DNMT1.

Supplementary Table 3: Detail of the training and test dataset including number of compounds and bio-activity values.

|  | **Total no. of Compounds** | **Actives** | **Least-Actives** | **Activity Range**  IC_50_ μM | **Threshold**  IC_50_ μM |
| --- | --- | --- | --- | --- | --- |
| **Training Set** | 196 | 146 | 50 | 0.03-1600 | 10 μM |
| **Test Set** | 46 | 36 | 10 | 0.01-132 | 10 μM |

Supplementary Table 4: Description of two dimensional features (MOE) that were utilized for the development of ML models.

| **2D Descriptors** | **Description** |
| --- | --- |
| vsa_acc | Approximated sum of VDW surface area of hydrogen bond acceptors |
| h_logP | octanol-water partition coefficient |
| b_count | Number of total bonds |
| radius | Measure of radius |
| PEOE_VSA-5 | Partial-charges based moe descriptors calculated on the basis of electro-negativities where *qi* range [-0.30,-0.25] (Gasteiger and Marsili 1980) |
| b_double | No of double bonds (aromatic are exempted) |
| SlogP_VSA5 | MOE descriptors using surface area and SlogP (0.15, 0.20) contribution (Wildman and Crippen 1999) |
| SMR_VSA2 | MOE descriptors using surface area & molar refractivity (0.26, 0.35) contribution |
| Q_VSA_PNEG | Total negative polar van der Waals surface area. This is the sum of *vi* such that *qi* is less than –0.2. The *vi* are calculated using a connection table approximation. (Gasteiger and Marsili 1980) |
| Kier 3 | Third alpha modified shape index |


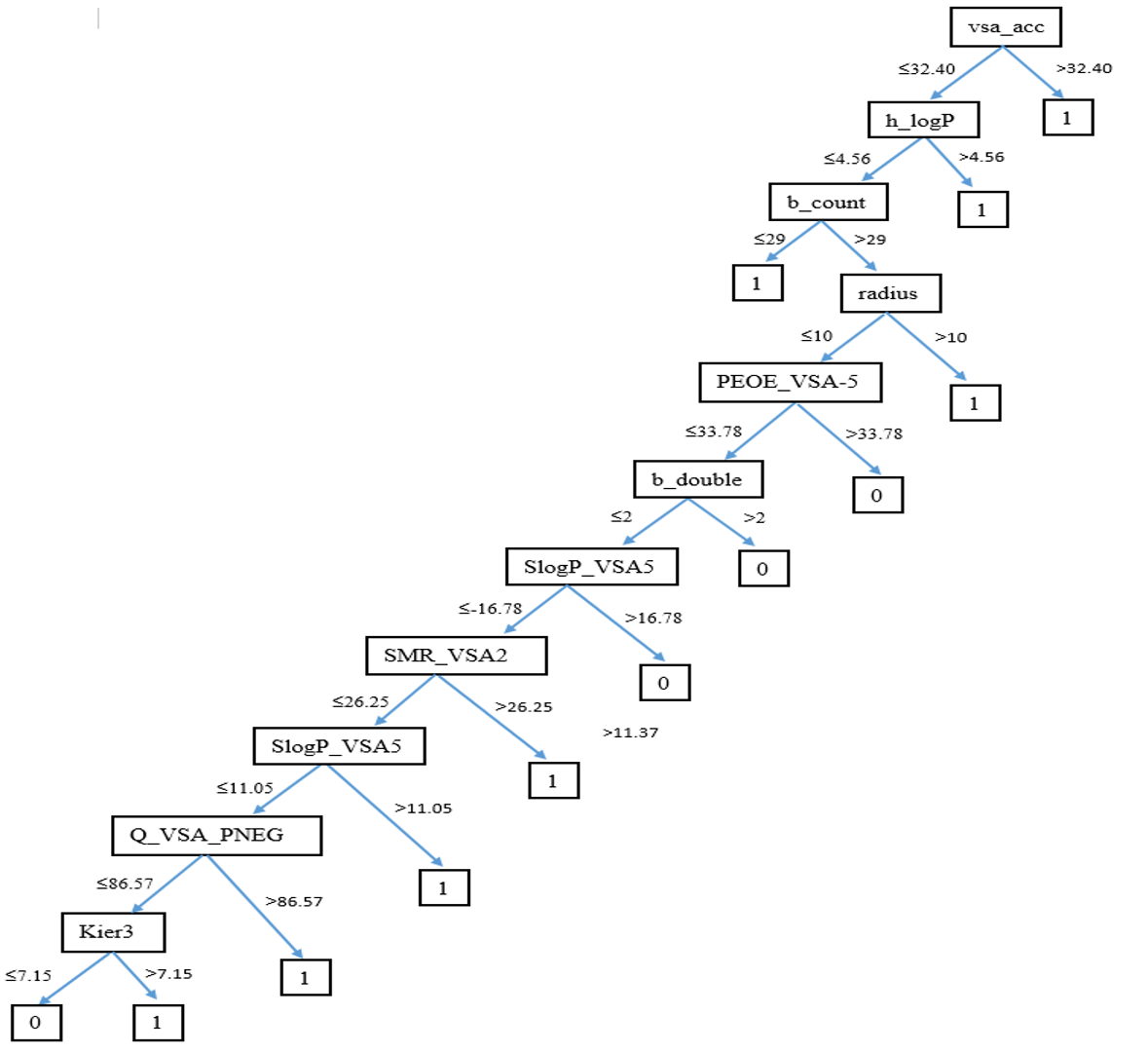


Supplementary Figure 2: Topology of pruned J-48 decision tree generated using C4.5 WEKA software is presented here. The tree size is 23 with a total of 12 terminal nodes where internal nodes represent descriptors along with threshold values that lead to the classification into active (1) or least-active (0) class label.


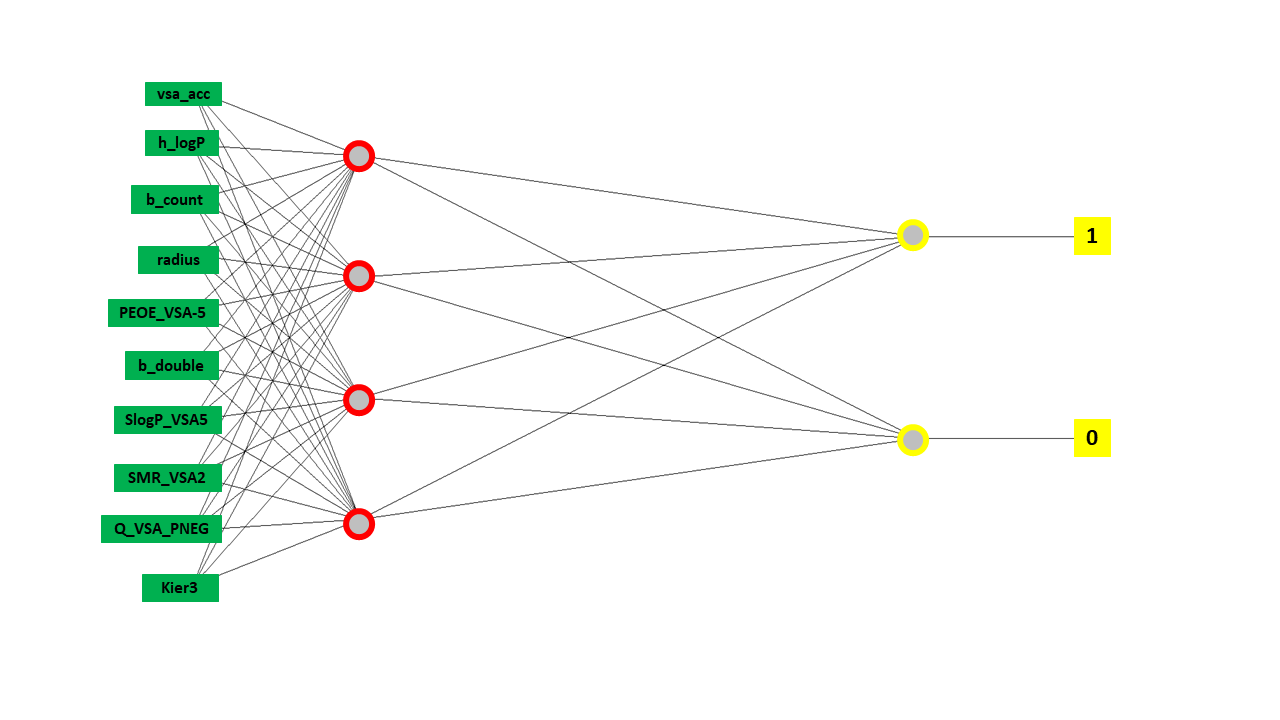


**Supplementary Figure 3:** **Multilayer perceptron neural network that was trained with 10 inputs (green nodes) and 4 hidden nodes (red nodes) is presented here. Each green color box represents an input node defined by the attributes whereby red circles depict number of hidden layers. Output nodes are represented in yellow color followed by decision node with class label (1= active, 0= least active).**
